# The checkpoint protein Zw10 connects CAL1-dependent CENP-A centromeric loading and mitosis duration in Drosophila cells

**DOI:** 10.1371/journal.pgen.1008380

**Published:** 2019-09-25

**Authors:** Anne-Laure Pauleau, Andrea Bergner, Janko Kajtez, Sylvia Erhardt

**Affiliations:** 1 Center for Molecular Biology of Heidelberg University (ZMBH), Heidelberg, Germany; 2 DKFZ-ZMBH-Alliance, Heidelberg, Germany; 3 CellNetworks Excellence Cluster, Heidelberg University, Heidelberg, Germany; Duke University, UNITED STATES

## Abstract

A defining feature of centromeres is the presence of the histone H3 variant CENP-A that replaces H3 in a subset of centromeric nucleosomes. In Drosophila cultured cells CENP-A deposition at centromeres takes place during the metaphase stage of the cell cycle and strictly depends on the presence of its specific chaperone CAL1. How CENP-A loading is restricted to mitosis is unknown. We found that overexpression of CAL1 is associated with increased CENP-A levels at centromeres and uncouples CENP-A loading from mitosis. Moreover, CENP-A levels inversely correlate with mitosis duration suggesting crosstalk of CENP-A loading with the regulatory machinery of mitosis. Mitosis length is influenced by the spindle assembly checkpoint (SAC), and we found that CAL1 interacts with the SAC protein and RZZ complex component Zw10 and thus constitutes the anchor for the recruitment of RZZ. Therefore, CAL1 controls CENP-A incorporation at centromeres both quantitatively and temporally, connecting it to the SAC to ensure mitotic fidelity.

## Author summary

Segregation of DNA during mitosis is a highly regulated process necessary to ensure the faithful transmission of genetic material to new daughter cells. Centromeric chromatin, which is defined by the presence of the histone H3 variant CENP-A, mediates the interaction of chromosomes with the spindle apparatus. In most organisms, CENP-A incorporation into centromeric chromatin is uncoupled from DNA replication and depends on specialized loading mechanisms and chaperones. In Drosophila cells, CENP-A loading takes place during mitosis and is mediated by its loading factor CAL1. We show that CAL1 controls the amount as well as the timing of CENP-A incorporation into centromeric chromatin. Moreover, CENP-A loading inversely correlates with the duration of mitosis. Mitosis length is influenced by the spindle assembly checkpoint, which ensures that all centromeres are attached correctly to the microtubule spindle. We identified the checkpoint protein and RZZ component Zw10 as a new interactor of CAL1. This interaction establishes a connection between CENP-A loading and mitosis control. We hypothesize that this crosstalk between CENP-A loading and spindle assembly checkpoint ensures that mitosis does not proceed until a sufficient amount of CENP-A is incorporated, thereby safeguarding centromere function and chromosome segregation.

## Introduction

The formation of two genetically identical daughter cells with a correct and stable genome is of utmost importance during mitosis. Condensed chromosomes are attached and segregated to the opposing poles of the dividing cell at anaphase by the mitotic spindle. At the interface between the chromosomes and the spindle microtubules lies the kinetochore. This multi-protein complex is formed by the components of the KMN network (formed by the Knl1 complex, the Mis12 complex, and the Ndc80 complex) [1]. The Ndc80 complex is mainly responsible for connecting microtubules with kinetochores while the Knl1 complex primarily coordinates the Spindle Assembly Checkpoint (SAC) [2]. The SAC delays entry into anaphase until all chromosomes are properly attached and aligned at the metaphase plate. The metaphase to anaphase transition is controlled by the activation of Cdc20 of the APC/C, a multisubunit ubiquitin ligase that triggers the degradation of cell cycle regulators by the proteasome [3]. The SAC proteins Mad2, BubR1, and Bub3 sequester Cdc20 by forming the Mitotic Checkpoint Complex (MCC) thereby preventing the activation of the APC/C. Besides, other proteins have been implicated in SAC activity including Bub1, Mad1, the Mps1 and Aurora B kinases, and the RZZ complex (formed by the three proteins Rough Deal (ROD), Zw10 and Zwilch) [4]. Finally, the Mis12 complex serves as a hub at the kinetochore interacting with all kinetochore complexes as well as with the centromere [2].

The kinetochore assembles on the centromere during mitosis, a highly specialized chromatin region that is defined by an enrichment of nucleosomes containing the histone H3 variant CENP-A, also called CID in Drosophila. In contrast to canonical histones, CENP-A deposition at centromeres is independent of DNA replication and is temporally restricted to a specific cell cycle stage, which varies between organisms: late telophase/early G1 in mammalian cultured cells [5], G2 in S. pombe and plants [6–8], and mitosis to G1 in Drosophila [9–12]. The timing of CENP-A is particularly intriguing in Drosophila cultured cells as centromeric CENP-A is replenished during prometaphase-metaphase thus coinciding with kinetochore assembly. CENP-A loading requires the action of its dedicated chaperones: HJURP in humans, Scm3 in fungi and CAL1 in Drosophila [13–19]. Deregulation of CENP-A and its loading machinery can result in the misincorporation of CENP-A into regions along the chromosome arms [13–19]. Misincorporated CENP-A is usually rapidly degraded [20–22]. If, however, CENP-A-containing nucleosomes remain at non-centromeric sites ectopic formation of functional kinetochores can occur that may lead to chromosome segregation defects and aneuploidy [23–25].

In *Drosophila melanogaster*, two other proteins are constitutively present at centromeres and essential for centromere function: the conserved protein CENP-C [26, 27] and the CENP-A chaperone CAL1 (10, 17, 19). CENP-C has been shown to act as a linker between CENP-A nucleosomes and the Mis12 complex, therefore, providing a platform for kinetochore assembly [28]. CENP-C is also implicated in CENP-A replenishment at centromeres during mitosis by recruiting CAL1 [17, 19]. CAL1 interacts with CENP-A in both pre-nucleosomal and nucleosomal complexes [17] and is necessary for CENP-A protein stabilization via Roadkill-Cullin3-mediated mono-ubiquitination [29]. Moreover, CAL1 has been previously shown to be the limiting factor for CENP-A centromeric incorporation in fly embryos [30]. However, differences in centromere assembly have been reported between Drosophila cultured cells and embryos. Firstly, CENP-A loading has been shown to take place during mitotic exit in early embryos [9] and prometaphase to early G1 in cultured cells [10, 12]. Second, CENP-C incorporates concomitantly to CENP-A in embryos [9] while this time window seems to be larger in cultured cells [10]. We, therefore, set out to determine more precisely the function of CAL1 in CENP-A loading regulation in Drosophila cultured cells.

During the course of these studies, we found that overexpression of CAL1 not only increases endogenous and exogenous CENP-A abundance at centromeres, it also uncouples CENP-A loading from mitosis. Strikingly, we discovered a co-dependence of mitotic duration and accurate CENP-A loading that may be coordinated by an interaction of the CENP-A loading machinery with the SAC protein and RZZ subunit Zw10. These data suggest an intricate coordination of the spindle assembly checkpoint, CENP-A loading, and mitotic duration in order to safeguard accurate mitotic progression.

## Results

### CAL1 levels regulate the amount of CENP-A at centromeres

**Fig 1:**
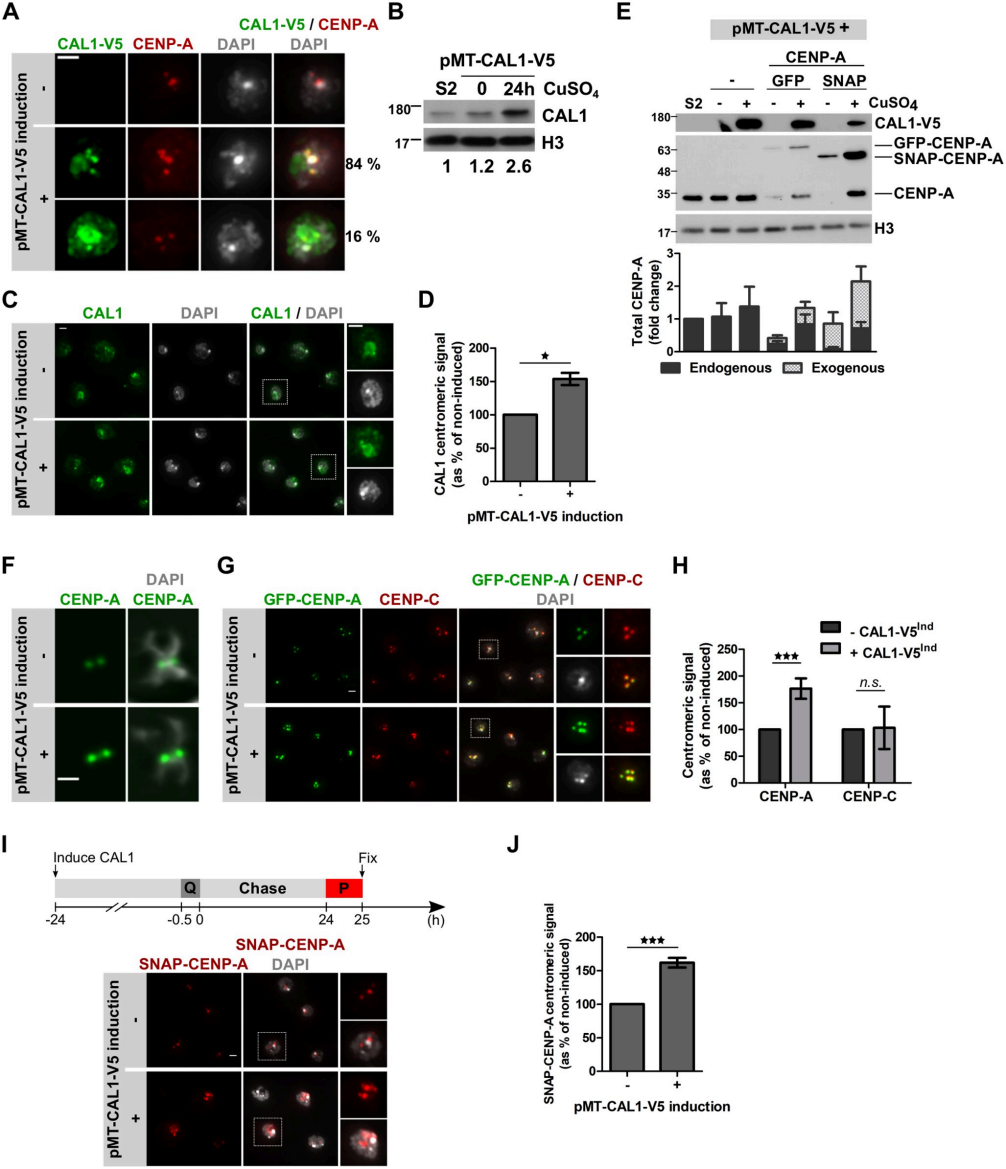
CAL1 overexpression leads to increased CENP-A levels at centromeres. **A**. Immunofluorescence of pMT-CAL1-V5-overexpressing cells. Expression was induced with 100 μM CuSO_4_ for 24 h (+). Controls are non-induced pMT-CAL1-V5 cells (-). Fixed cells are stained with anti-V5 antibody (green) and anti-CENP-A (red). DNA (DAPI) is shown in grey. The percentage of depicted localization patterns are indicated. **B**. Immunoblot of pMT-CAL1-V5-overexpressing cells treated as in A with anti-CAL1 (endogenous CAL1 and CAL1-V5) and anti-H3 antibodies. **C**. Immunofluorescence of pMT-CAL1-V5-overexpressing cells as in A stained with anti-CAL1 antibodies (green). DNA (DAPI) is shown in grey. **D**. Quantification of C showing CAL1 centromeric signal intensity per nucleus as % of non-induced control cells. **E**. Immunoblot of CENP-A (either GFP or SNAP N-terminally tagged, under the Copia promoter) expressing cells with or without concomitant pMT-CAL1-V5 induction. Anti-V5 (CAL1-V5), anti-CENP-A (endogenous CENP-A and tagged-CENP-A), and anti-H3 antibodies were used. Fold change of CENP-A levels compared to S2 cell are shown below (N = 4). **F**. Metaphase chromosomes of pMT-CAL1-V5-overexpressing cells as in A stained with anti-CENP-A antibody. DNA (DAPI) is shown in grey. Scale bar: 1 μm. **G**. Immunofluorescence of pMT-CAL1-V5/GFP-CENP-A expressing cells as in A stained with anti-CENP-C antibody (red). DNA (DAPI) is shown in grey. **H**. Quantification of CENP-A and CENP-C signal intensities per centromere as shown in G. **I**. Timeline of the Quench-Chase-Pulse SNAP-tag experiment after 24 h pMT-CAL1-V5 induction: cells were incubated with SNAP-Block to quench existing SNAP-CENP-A molecules (Q), washed and cultured for 24 h (chase), newly synthesized SNAP-CENP-A molecules were stained with SNAP-SiR647 (P). Representative images of SNAP-CENP-A (red) in non-induced and induced pMT-CAL1-V5 cells. DNA (DAPI) is shown in grey. **J**. Quantification of I. SNAP-CENP-A signal intensity per centromere shown as % of non-induced control. Scale bar: 2 μm. All graphs show Mean +/- SEM of 3 experiments (n>300 cells), Student’s t-test (*n*.*s*.: non-significant; *: p<0.05; **: p<0.01, ***: p<0.001).

CAL1 is essential for CENP-A loading but we do not fully understand its role in restricting CENP-A loading to defined cell cycle stages. To investigate how CAL1 influences CENP-A loading, we overexpressed CAL1 in S2 cells from a copper-inducible metallothionein (MT) promoter. Induced CAL1 localized to centromeres and the nucleolus in a majority of transfected cells (84%), similar to endogenous CAL1 [13–19]. Additional cytoplasmic and/or nucleoplasmic CAL1 was visible in about 16% of cells (Fig 1A). Augmented total CAL1 protein levels (2.6-fold increase; Fig 1B) led to an increased CAL1 level at centromeres (Fig 1C and 1D) and caused an increase of both endogenous and exogenous, tagged CENP-A (GFP and SNAP) levels (Fig 1E). Interestingly, endogenous CENP-A levels were downregulated in cell lines that expressed an exogenous CENP-A construct, independent of CAL1 levels (Fig 1E), indicating that cells sense the overall level of CENP-A and either degrade excess CENP-A (possibly by already described proteasomal degradation mechanisms) or cannot stabilize CENP-A due to limiting amounts of CAL1 (and CAL1-dependent mono-ubiquitination of CENP-A) [22, 29, 31]. Importantly, however, CENP-A localized exclusively to centromeres in CAL1-overexpressing cells, and we did not detect CENP-A at any other chromatin regions on metaphase chromosomes under these conditions (Fig 1F). This result indicates that CAL1 overexpression does not lead to the misincorporation of CENP-A to non-centromeric sites that has been observed in CENP-A overexpression systems [13–19]. In contrast to previous observations in embryos where increased centromeric CENP-A levels required both CAL1 and CENP-A overexpression [30], we observed in cells that endogenous centromeric CENP-A levels were increased by up to 2 fold after 24 hours of CAL1 overexpression (Fig 1G and 1H). Furthermore, CENP-C levels remained largely unaffected by CAL1 overexpression (Fig 1G and 1H). These results show that the abundance of CENP-A at centromeres depends on the available amount of CAL1.

To test whether the increased CENP-A level is caused by increased CENP-A loading we used the SNAP-tag technology in a quench-chase-pulse experiment to label newly synthesized and incorporated CENP-A [5]. At day 1 of CAL1 induction, we quenched existing SNAP-CENP-A molecules with a non-fluorescent ligand (SNAP-Block), washed out unbound Block, cultured the cells for 24 hours to allow approximately one additional cell division, then marked newly synthesized SNAP-CENP-A molecules with a fluorophore, and measured the fluorescent intensity of newly incorporated SNAP-CENP-A at centromeres (Fig 1I). Similar to what we observed for GFP-CENP-A, significantly more SNAP-CENP-A incorporated into centromeres in CAL1-overexpressing cells (Fig 1I and 1J). These data confirm that CENP-A centromeric levels are regulated by CAL1 and that CAL1 overexpression specifically increases the incorporation of newly synthesized CENP-A at centromeric chromatin.

### Overexpression of CAL1 leads to centromeric CENP-A loading outside of mitosis

**Fig 2:**
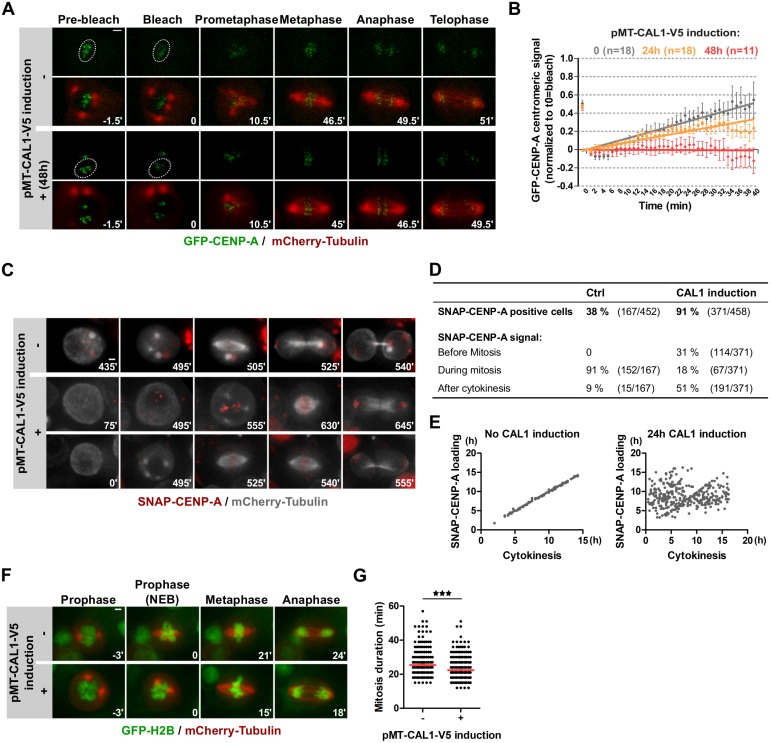
Overexpression of CAL1 leads to CENP-A centromeric loading outside of mitosis. **A**. FRAP experiments of GFP-CENP-A in pMT-CAL1-V5-overexpressing cells during mitosis. After 48 h pMT-CAL1-V5 induction, GFP-CENP-A signal was partially bleached in prophase and cells imaged until telophase. Time-lapse: 90 s. Scale bar: 2 μm. **B**. Quantification of A. The total GFP-CENP-A centromeric signal is shown as mean +/- SEM, n ≥ 11 cells. **C**. Time-lapse imaging of SNAP-CENP-A/mCherry-Tubulin cells with or without prior induction of pMT-CAL1-V5 (24 h). Cells were treated with SNAP-Block to quench existing SNAP-CENP-A molecules and washed before adding 0.5 μM SNAP-640 dye to visualize newly synthesized SNAP-CENP-A. Imaging: 16 h. Time-lapse: 15 min. Scale bar: 2 μm. Intensity levels have been adjusted separately for each condition. **D-E**. Quantifications of C. **D**. Percentage of SNAP-CENP-A positive cells and their timing of loading. **E**. For each cycling cell, the earliest detection time point of SNAP-CENP-A (Y-axis) is plotted versus the time of cytokinesis (X-axis). t0 on both axes corresponds to the start of imaging after SNAP-Block. **F**. Time-lapse imaging of H2B-GFP/mCherry-Tubulin cells with or without prior induction of pMT-CAL1-V5 (24 h). Imaging: 16 h. Time-lapse: 3 min. Scale bar: 2 μm. **G**. Quantification of F showing the mitosis duration from nuclear envelope breakdown (determined by mCherry-Tubulin nuclear diffusion concomitant to DNA condensation) to anaphase entry. Mean +/- SEM, n>200 cells. Student’s t-test (*: p<0.05; **: p<0.01, ***: p<0.001).

CENP-A loading takes place during prometaphase-metaphase in Drosophila S2 cells [10]. To determine if CAL1 overexpression changes the CENP-A loading pattern during mitosis, we measured Fluorescence Recovery After Photobleaching (FRAP) of GFP-CENP-A, on the assumption that fast recovery of the signal at centromeres corresponds to active loading of CENP-A [12]. We partially bleached the GFP-CENP-A signal during prophase and followed the recovery of the signal at centromeres until anaphase. We observed a partial recovery of GFP-CENP-A at centromeres in control cells, which could be due to either turnover of unbleached GFP-CENP-A or to the loading of new molecules. This is in agreement with previous reports that the loading of centromeric CENP-A takes place during mitosis [10] (Fig 2A and 2B). Surprisingly, the GFP-CENP-A recovery rate decreased in CAL1-overexpressing cells compared to control cells (Fig 2B), suggesting that the maximal level of CENP-A at centromeres has been reached at the time of the bleaching event. One possible explanation could be that CENP-A centromeric levels get replenished at a different cell cycle stage in CAL1-overexpressing cells. Indeed, besides loading in mitosis, CENP-A loading has also been reported in G1 in S2 cells [12]. We, therefore, followed cells through mitosis, partially bleached GFP-CENP-A in early G1 and measured GFP-CENP-A signal intensity for 3 hours. However, we did not detect any recovery of the bleached GFP-CENP-A signal (S1A Fig). We explained these results by the likely absence of any measurable GFP-CENP-A loading in early G1. This also indicates that centromeric CENP-A has a very low turnover, at least during G1.

Since the availability of CAL1 seems to be the crucial determinant for CENP-A incorporation, we tested if CAL1-overexpressing cells incorporate CENP-A into centromeric chromatin outside mitosis. Thus, we arrested cells at the metaphase to anaphase transition using the proteasome inhibitor MG132 for 2 hours, blocked existing SNAP-CENP-A molecules using SNAP-Block, and cultured the cells for an additional 4 hours in the presence of MG132 (S1B Fig) similar to a previously published protocol [10]. The MG132 treatment prevents cells from exiting mitosis, which means that every SNAP-CENP-A positive interphase cell must have acquired SNAP-CENP-A outside of mitosis. While 10% of uninduced pMT-CAL1 cells loaded SNAP-CENP-A, the induction of pMT-CAL1 and resulting overexpression of CAL1 led to incorporation of newly synthesized SNAP-CENP-A in about 40% of the cells, pointing to loading outside of mitosis (S1B Fig). To confirm that overexpressed CAL1, indeed, loads CENP-A to centromeres outside of mitosis, we followed newly synthesized SNAP-CENP-A live by time-lapse microscopy. We blocked existing SNAP-CENP-A with SNAP-Block, washed and added SNAP-640 fluorophores [32] to the culture medium to fluorescently mark newly synthesized SNAP-CENP-A molecules. Despite some aggregation of the dye, SNAP-CENP-A loading at centromeres was discernible in both CAL1-overexpressing and control cells (Fig 2C). In this assay, we only analyzed cells that were actively cycling and eventually entered mitosis during the course of imaging, and determined whether and when they incorporated SNAP-CENP-A. As a reference point, we used the first time point where the intercellular microtubule bridge was visible (cytokinesis). We found that 38% of uninduced control cells had SNAP-CENP-A signals at centromeres by the end of the imaging (Fig 2C–2E; S1 Video). Of those, 91% loaded SNAP-CENP-A during mitosis and 9% just after the cell exited mitosis. In contrast, 81% of cycling CAL1-overexpressing cells had SNAP-CENP-A signals at centromeres (Fig 2C–2E; S2 Video), and of that 31% loaded before mitosis and 51% after the cells exited mitosis. The specificity of the signal was verified by co-staining with anti-CENP-A antibody immediately after block (0 min), or after 20 hours of incubation with SNAP-640 (S1C Fig). No SNAP-CENP-A was detected directly after SNAP-block treatment, confirming the efficient quenching of old SNAP-CENP-A molecules (S1C Fig). After 20 hours newly synthesized SNAP-CENP-A perfectly co-localized with total CENP-A signals at centromeres (S1C Fig). Interestingly, we did not observe any differences in the percentage of cells with SNAP-CENP-A signal at centromeres between control and CAL1-overexpressing cells after 20 h (S1D Fig). However, the intensity of SNAP-CENP-A at centromeres was four times higher in CAL1-overexpressing cells than in control cells (S1E Fig). These results are not consistent with the live analysis of the same cell line (Fig 2C and 2D). This discrepancy is probably due to the masking of low levels of SNAP-CENP-A in the presence of excess SNAP dye in live cell analysis (Fig 2D). Nevertheless, we can conclude that cells overexpressing CAL1 load CENP-A throughout the cell cycle, while loading outside mitosis does not occur in control cells (Fig 2D and 2E) suggesting that CAL1 misexpression uncouples CENP-A centromeric loading from mitosis and leads to an overload of CENP-A at centromeres.

We next analyzed CAL1-overexpressing cells live to determine the consequences of increased CENP-A centromeric levels on mitosis progression. We performed time-lapse microscopy experiments in cells overexpressing CAL1 together with H2B-GFP and mCherry-Tubulin to mark the chromatin and mitotic spindle, respectively. Surprisingly, we found that mitosis duration, defined as the time between partial nuclear envelope breakdown (NEB) and anaphase entry, was 10% shorter in CAL1-overexpressing cells as compared to control cells (Fig 2F and 2G; S3 and S4 Videos). We also measured mitosis duration in cells containing GFP-CENP-A and inducible CAL1, which showed similar results (S2A and S2B Fig). Of note, CAL1 overexpression caused a slight but significant increase of lagging chromosomes (S2C Fig). We also scored cells where mitosis was generally defective (multipolar spindles, micronuclei formation) but did not measure an increase in CAL1-overexpressing cells (S2D Fig), suggesting that the observed lagging chromosomes resolved themselves before mitotic exit. Kinetochore proteins (CENP-C, Spc105R, GFP-Zw10) were equally recruited during prometaphase when we compared CAL1-overexpressing cells with non-induced cells (S2E–S2G Fig). To determine if kinetochores attachment to the spindle microtubules was affected in CAL1-overexpressing cells, we performed an MG132-Taxol assay as described in Maia et al., [33]. This assay allowed us to score the attachment status of each kinetochore, which we found equal in control and CAL1-overexpressing cells (S2H Fig).

Last but not least, we ought to determine if the SAC activity was affected by increased centromeric CENP-A levels. Similar amounts of BubR1 and GFP-Mad2 were recruited in CAL1-overexpressing prometaphase cells when compared to non-induced cells (S3A–S3B Fig) and the SAC was functioning normally since cells treated with the microtubule interfering drug Taxol triggered a cell cycle arrest in prometaphase in control cells as well as in CAL1-overexpressing cells (S3C and S3D Fig). However, CAL1-overexpressing cells spend less time in an arrested state before reverting to an interphase state without performing cytokinesis indicating that SAC activity is weakened in the presence of excess CENP-A at centromeres (S3E Fig). We concluded that CAL1-overexpressing cells not only incorporated more CENP-A at centromeres, they also progressed faster through mitosis with comparable kinetochore formation and SAC activity. We, therefore, wondered if CENP-A levels regulate mitosis duration.

### CENP-A levels correlate with mitosis duration

**Fig 3:**
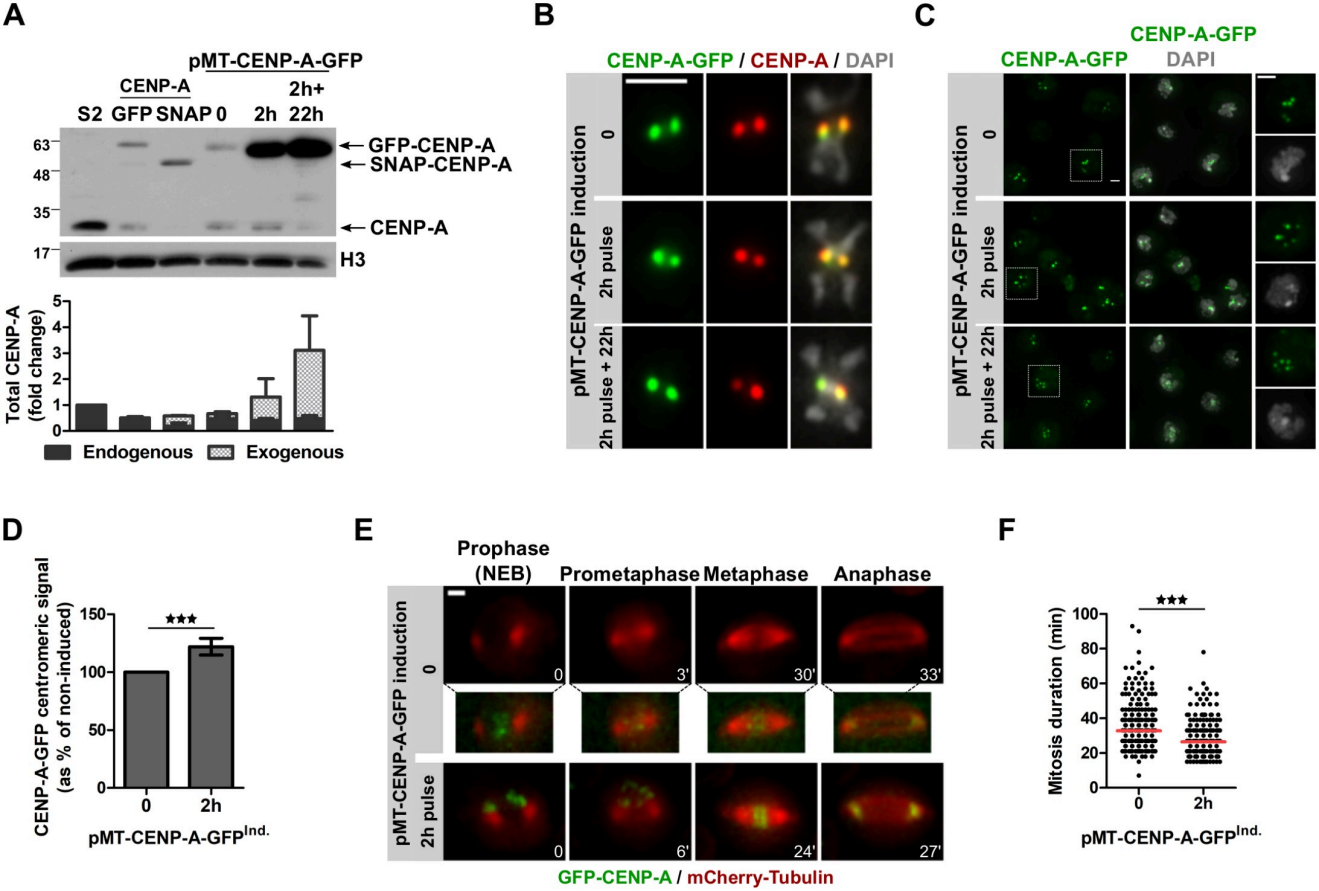
CENP-A overexpression is associated with shorter mitosis duration. **A**. Immunoblot showing CENP-A levels in different cell lines. CENP-A antibodies detect endogenous and tagged CENP-A. GFP-CENP-A and SNAP-CENP-A are under the constitutive *Copia* promoter; CENP-A-GFP was induced with 10 μM CuSO_4_ for 2 h. H3 serves as a loading control. The graph shows the fold change of CENP-A compared to S2 cells (N = 4). **B**. Metaphase chromosomes of pMT-CENP-A-GFP cells induced with 10 μM CuSO_4_ for 2 h stained with anti-CENP-A antibody. DNA (DAPI) is shown in grey. Intensities have been adjusted for each condition. Scale bar: 2 μm. **C**. Immunofluorescence of pMT-CENP-A-GFP cells as in B. DNA (DAPI) is shown in grey. Scale bar: 2 μm. **D**. Quantification of C showing the total CENP-A-GFP centromeric intensity per nucleus as % of non-induced pMT-CENP-A-GFP. Mean +/- SEM of 3 experiments (n>300 cells), Student’s t-test (***: p<0.001). **E**. Time-lapse imaging of cells expressing mCherry-tubulin and pMT-CENP-A-GFP induced as in B, washed, and imaged for 16 h. Time-lapse: 3 min. Scale bar: 2 μm. The intensity of CENP-A-GFP in control cells is enhanced for visualization purposes. **F**. Quantification of mitosis duration shown in E. Mean +/- SEM, n>300 cells. Student’s t-test (***: p<0.001).

First, we investigated if elevated CENP-A levels alone (without CAL1 overexpression) would influence mitosis duration. However, upregulating CENP-A to high levels is associated with ectopic CENP-A incorporation into chromosome arms and subsequent defects in mitosis [23]. To avoid these detrimental effects, we induced CENP-A overexpression from a pMT-CENP-A-GFP plasmid with a short and very low CuSO_4_ pulse (2 hours; 10 μM CuSO_4_). Under those conditions, CENP-A-GFP was induced while endogenous CENP-A levels were decreased leading to an overall relative increased CENP-A level (Fig 3A). 22 hours after the CuSO_4_ pulse, untagged CENP-A protein was almost undetectable by Western blot, suggesting that cells control the total abundance of CENP-A protein levels to some degree (Fig 3A), probably through proteasomal degradation as reported before [22, 23, 31]. This compensation of CENP-A protein amount was detectable also in cells constitutively expressing GFP-CENP-A or SNAP-CENP-A from the copia promoter (Fig 3A). Under these mild induction conditions, non-centromeric CENP-A or CENP-A-GFP were undetectable (Fig 3B) and an increased amount of CENP-A-GFP and CAL1 localized to centromeres (Fig 3C and 3D and S4A Fig). Using time-lapse analysis, we found that cells with increased centromeric CENP-A levels progressed significantly faster through mitosis (Fig 3E and 3F; S5 and S6 Videos) without increased segregation defects (S4B Fig). Similar to CAL1-overexpressing cells, CENP-A-overexpressing cells did not recruit more kinetochore proteins during mitosis (Ndc80; S4C Fig) nor did they show any defects in kinetochore-microtubules attachments (S4D Fig).

**Fig 4:**
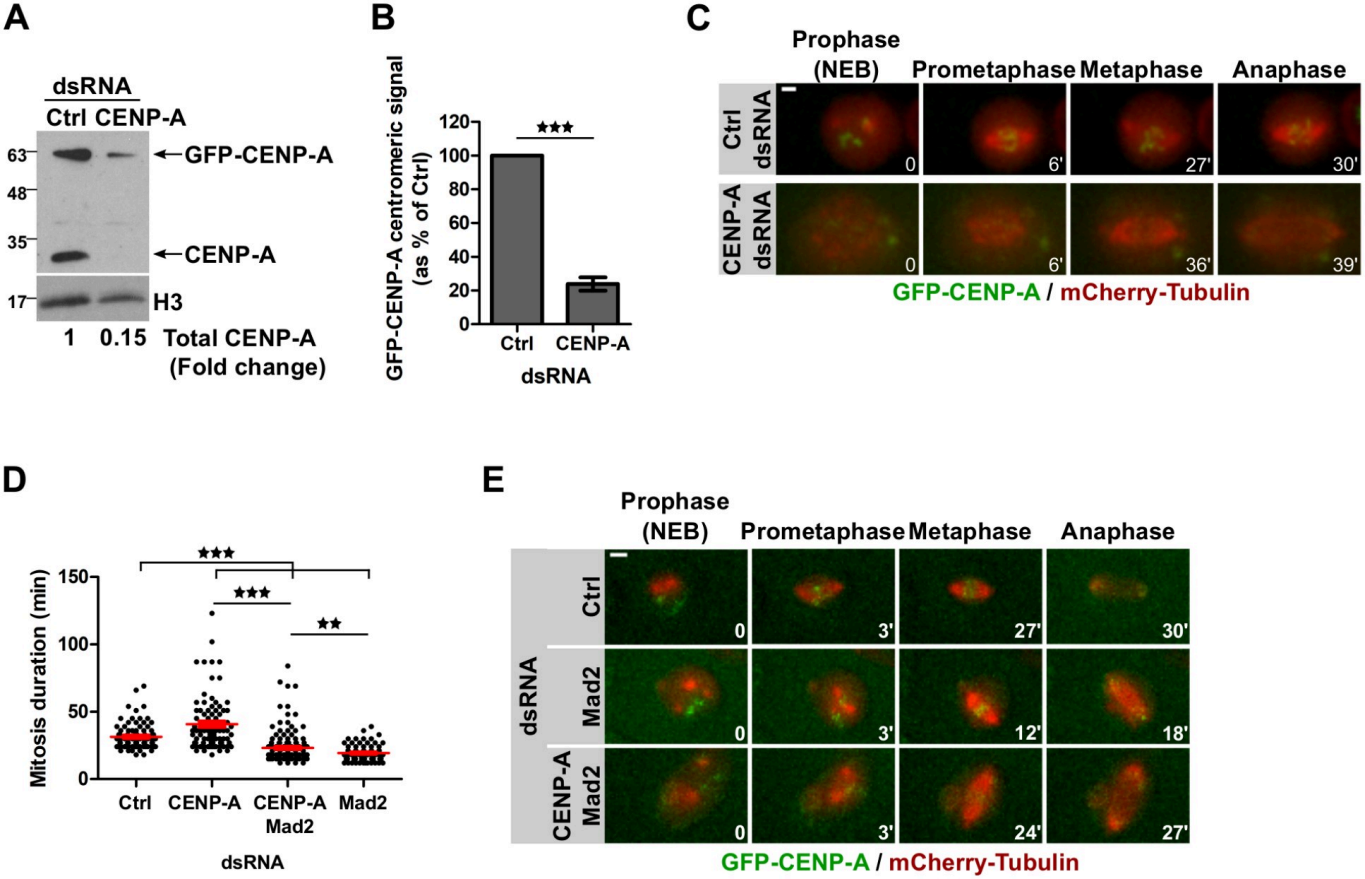
Reduced CENP-A at centromeres leads to longer mitosis through SAC activity. **A**. Immunoblot showing CENP-A knockdown efficiency (72 h) in GFP-CENP-A/mCherry-Tubulin expressing cells using anti-CENP-A antibodies to detect endogenous CENP-A and overexpressed GFP-CENP-A. **B**. Quantification showing GFP-CENP-A centromeric signal intensity per nucleus at t0 of time-lapse imaging as in C. Mean +/- SEM, n>80 cells. Student’s t-test (***: p<0.001). **C**. Time-lapse imaging of GFP-CENP-A/mCherry-tubulin expressing cells after 72 h CENP-A depletion. Imaging: 16 h. Time-lapse: 3 min. Scale bar: 2 μm. **D**. Quantification of C and E showing the mitosis duration of control, CENP-A, Mad2 or CENP-A/Mad2-depleted cells. Mean +/- SEM, n>80 cells. Student’s t-test (***: p<0.001). **E**. Time-lapse imaging of GFP-CENP-A/mCherry-tubulin expressing cells after 72 h Mad2 or CENP-A/Mad2 depletion. Imaging: 16 h. Time-lapse: 3 min. Scale bar: 2 μm.

To further test the relationship between centromeric CENP-A abundance and the duration of mitosis we designed a strategy to reduce CENP-A levels without inducing chromosome alignment defects that might arrest cells in mitosis [34]. We performed CENP-A RNAi depletion in GFP-CENP-A-expressing cells, which led to undetectable levels of endogenous CENP-A while small amounts of GFP-CENP-A remained (Fig 4A and 4B). Mitosis duration was significantly extended in partially CENP-A-depleted cells when compared to control cells (Fig 4C and 4D; S7 and S8 Videos), probably due to defects or delays in kinetochore assembly and spindle attachment. Similar observations have been reported in heterozygous CENP-A mutant fly embryos [35], supporting our hypothesis that CENP-A levels control mitotic length. Partial co-depletion of the SAC protein Mad2 and CENP-A abrogates the mitotic delay observed in CENP-A-depleted cells indicating that the SAC is active in these cells (Fig 4D and 4E, S5A Fig). Taken together, these results further suggest that centromeric CENP-A levels influence mitosis duration in a SAC-dependent manner.

### CENP-A loading correlates with mitosis duration

**Fig 5:**
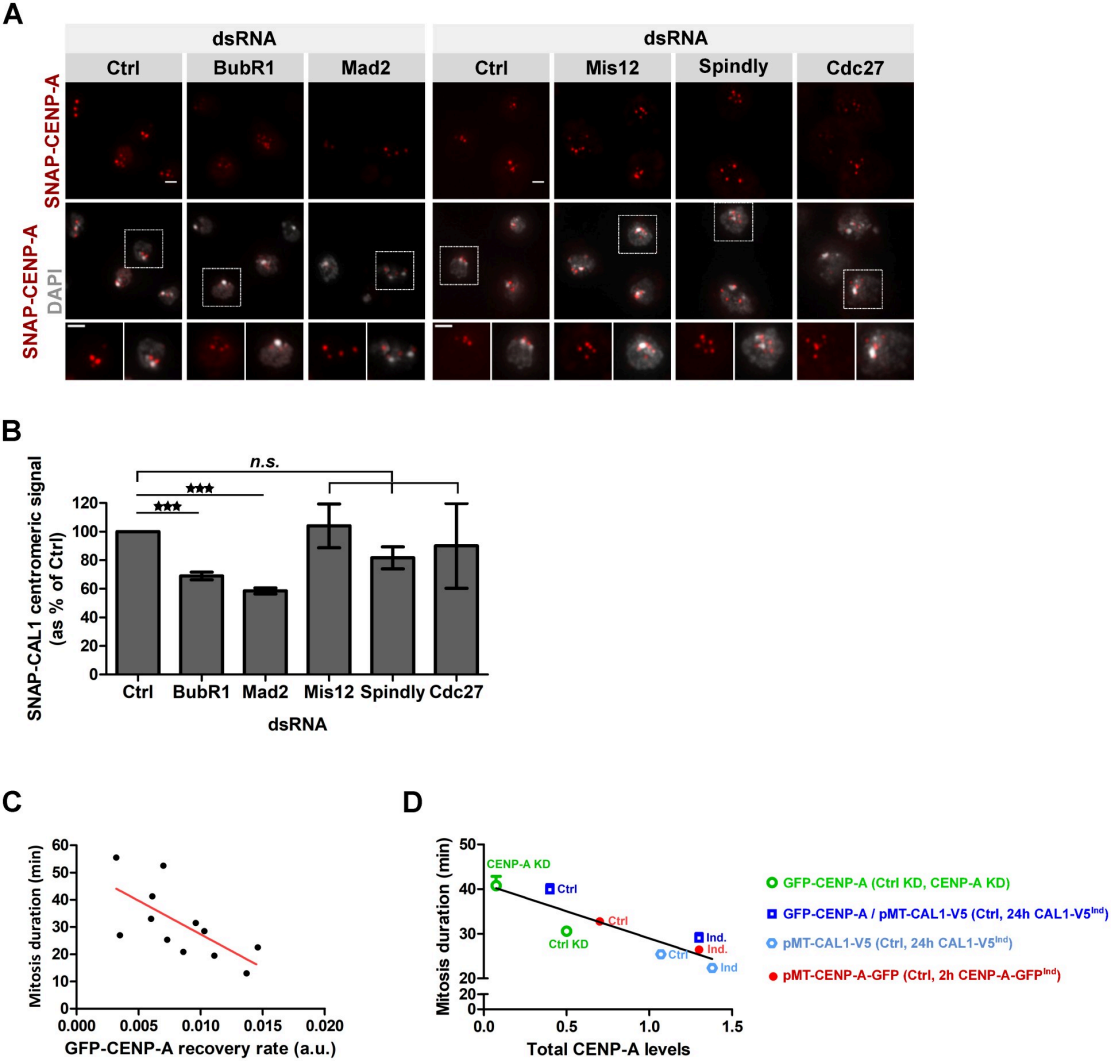
CENP-A loading is correlated with mitosis duration. **A**. SNAP-CENP-A incorporation in BubR1, Mad2, Mis12, Spindly or Cdc27-depleted cells. After 72 h dsRNA treatment, a Quench-Chase-Pulse experiment (scheme in Fig 1H) was performed to stain newly synthesized SNAP-CENP-A molecules (red). DNA (DAPI) is shown in grey. Scale bar: 2 μm. **B**. Quantification of A. The total SNAP-CENP-A centromeric signal intensity per nucleus is shown as % of control cells. Mean +/- SEM of 3 experiments (n>300 cells), Student’s t-test (*n*.*s*.: non-significant; ***: p<0.001). **C**. The recovery rate of GFP-CENP-A after photobleaching in mitosis plotted against the mitosis duration for each cell. Pearson’s correlation test, p<0.01. **D**. Total CENP-A protein levels (determined by immunoblotting) are plotted against the mitosis duration for each cell line. Pearson’s correlation test, p<0.01.

CENP-A loading peaks during prometaphase-metaphase in Drosophila S2 cells. Therefore, we hypothesized that the amount of newly synthesized CENP-A that can be incorporated into centromeric chromatin depends on mitosis duration. Mitotic progression is controlled by the action of SAC proteins, which delay entry into anaphase until all chromosomes are properly attached and aligned at the metaphase plate. To shorten mitosis, we depleted the two mitotic checkpoint proteins, BubR1 and Mad2, known to reduce mitosis duration [36–38]. These depletions (S5B and S5C Fig) led to a decrease in newly synthesized centromeric SNAP-CENP-A incorporation (Fig 5A and 5B). In contrast, the depletion of Mis12 (S5C Fig), which has been previously shown not to affect the duration of mitosis in Drosophila cultured cells [39], did not alter SNAP-CENP-A amount at centromeres (Fig 5A and 5B). Extending metaphase duration by preventing entry into anaphase through depletion of Spindly (required for silencing of the SAC) [40] or of the APC/C cofactor Cdc27 [41](S5C Fig) did not cause an increase of CENP-A levels at centromeres when compared to control (Fig 5A and 5B). Knockdown efficiencies were determined by immunoblot and/or qPCR (S5B and S5C Fig). We further irreversibly arrested cells in prometaphase by the microtubule-depolymerizing drug Nocodazole or in metaphase by the proteasome inhibitor MG132. These conditions also did not change the amount of newly synthesized SNAP-CENP-A at centromeres (S5D and S5E Fig). SNAP-CAL1 centromeric levels were similarly affected: an acceleration of mitotic timing by BubR1 or Mad2 RNAi led to a decrease of loading, whereas mitotic arrest by Cdc27 or Spindly RNAi did not affect it (S5F Fig). This set of experiments suggests that the amount of new CENP-A molecules that are incorporated into centromeric chromatin is limited at each round of mitosis probably due to CAL1’s availability and that the length of mitosis is directly affected by the amount of centromerically loaded CENP-A. This anti-correlation can be seen best when we plotted the recovery rate of GFP-CENP-A measured by FRAP against the mitosis duration for each control cell that we measured (Fig 5C). This revealed that cells that incorporated GFP-CENP-A quickly progressed through mitosis faster than cells with a low recovery rate. To further illustrate this anti-correlation between CENP-A levels and mitosis duration, we plotted the mitosis duration versus CENP-A total protein levels determined by immunoblotting for all the cell lines used in this study (Fig 5D). This confirmed a clear anti-correlation of CENP-A levels and mitotic duration and further suggests that mitosis duration correlates with CENP-A centromeric levels rather than with the rate of CENP-A incorporation.

### RZZ component Zw10 interacts with centromeric proteins

**Fig 6:**
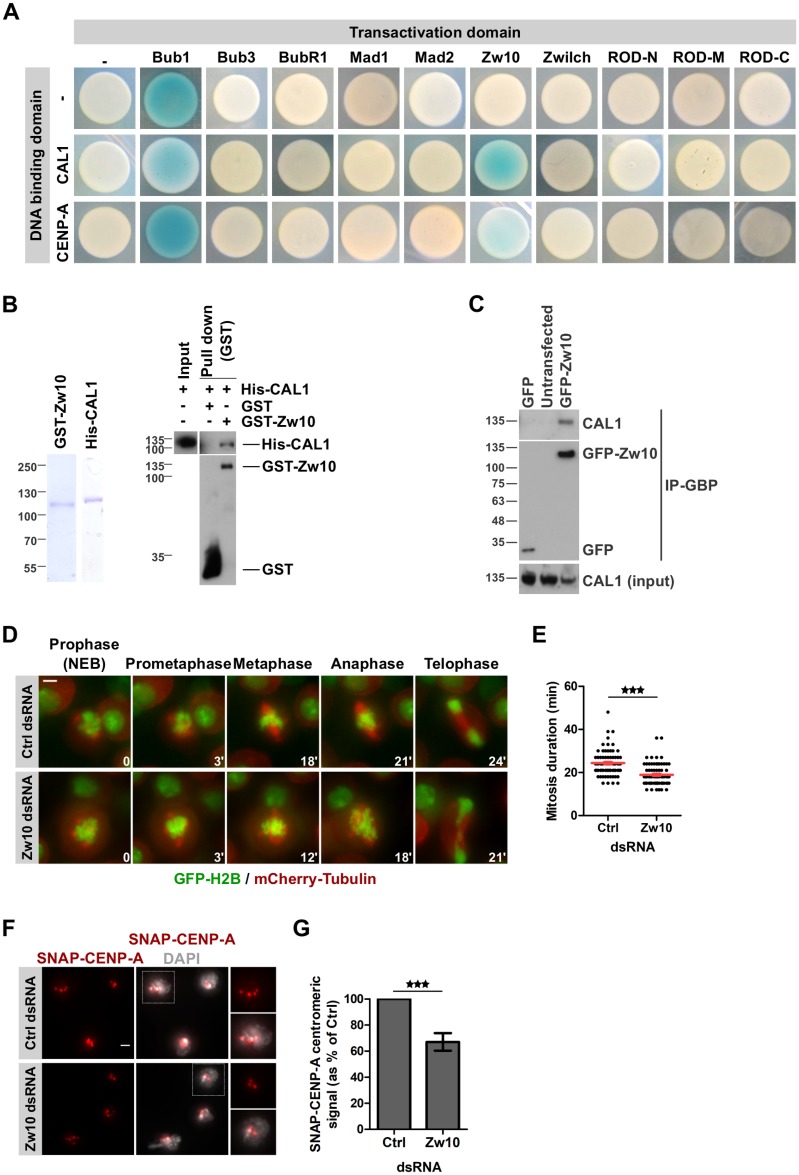
Zw10 interacts with CENP-A loading factor CAL1. **A**. Yeast two-hybrid interaction tests using SAC proteins as prey with either CAL1 or CENP-A as bait. The blue color indicates an interaction between prey and bait. **B. *Left panel***. Coomassie showing purified GST-Zw10 and His-CAL1. ***Right panel***. Immunoblot showing GST-pulldown assays of His-CAL1. **C**. Co-immunoprecipitation of CAL1 with GFP-Zw10 using the GFP-binding protein (GBP). Pulled down fractions were analyzed for the presence of CAL1. **D**. Time-lapse imaging of H2B-GFP/mCherry-tubulin expressing cells after 72 h Zw10 depletion. Imaging: 16 h. Time-lapse: 3 min. Scale bar: 2 μm. **E**. Quantification of mitosis duration shown in D. Mean +/- SEM, n>200 cells. Student’s t-test (***: p<0.001). **F**. SNAP-CENP-A incorporation in Zw10-depleted cells. After 72 h dsRNA treatment, a Quench-Chase-Pulse experiment (scheme in Fig 1H) was performed to stain newly synthesized SNAP-CENP-A molecules (red). DNA (DAPI) is shown in grey. Scale bar: 2 μm. **G**. Quantification of F showing the total SNAP-CENP-A centromeric intensity per nucleus as % of control. Mean +/- SEM of 3 experiments (n>300 cells), Student’s t-test (***: p<0.001).

In order to identify a putative connection between CENP-A loading and mitosis progression, we performed a targeted Yeast-two-hybrid (YTH) screen using either CAL1 or CENP-A as bait and known SAC proteins as prey. These experiments showed that the RZZ component Zw10 binds to full-length CAL1 and CENP-A (Fig 6A). To identify the domains of each protein required for the interaction, we repeated the YTH analysis with fragments of CAL1 and Zw10. This analysis revealed a strong interaction of Zw10 with the C-terminus of CAL1 (amino acids 680–979) (S6A Fig). We further identified Zw10 C-terminus (amino acids 482–721) as being sufficient to bind to the CAL1 C-terminus (S6A Fig), the same domain of CAL1 that also binds CENP-C and Roadkill (S6B Fig) [29, 30]. No further interactions were detected between the other checkpoint proteins and the centromeric proteins suggesting that Zw10 might be the key component that connects the centromere loading machinery to checkpoint proteins and ultimately mitosis duration. Our analysis regarding Bub1 has been inconclusive due to the self-activation of the Bub1 construct in this assay (blue color in the control (-) transformation). The absence of an interaction between CAL1 and Bub1 has, however, already been reported previously [30]. To validate the interaction between Zw10 and centromeric proteins, we expressed and purified GST-Zw10, His-CAL1, and His-CENP-A in bacteria. Pulldown experiments using those proteins confirmed that Zw10 directly binds CAL1 (Fig 6B). We did not detect any interaction between GST-Zw10 and His-CENP-A in this assay (S6C Fig). To further confirm the interaction between CAL1 and Zw10 in cells, we established a cell line expressing GFP-tagged Zw10. Firstly we confirmed that GFP-Zw10 localization in cultured cells (S6D Fig) is in accordance with endogenous protein [42–45] and transgene expression [46] reported in Drosophila tissues. Following the disassembly of the nuclear envelope at the onset of mitosis, Zw10 is recruited to the kinetochore where it remains until complete chromosome biorientation is reached. Zw10 is then transported along the spindle microtubules in a process mediated by Spindly and dynein, which is required to silence the SAC [40, 47–49]. We then performed immunoprecipitation experiments and co-immunoprecipitated CAL1 with GFP-Zw10 (Fig 6C), confirming our pull-down and YTH experiments. CENP-A was not consistently co-immunoprecipitated with Zw10, which we attributed to the fact that CENP-A interacts with CAL1 but probably not directly with Zw10. We concluded from this set of experiments that CAL1 robustly interacts with Zw10, and that this interaction may be involved in coordinating CENP-A centromeric loading with mitosis progression.

### Zw10 affects the loading of newly synthesized CENP-A during mitosis

The RZZ complex recruits the MAD proteins to the kinetochore and is thus essential for the SAC [46, 50]. Therefore, we sought to determine if Zw10 depletion could recapitulate the effects observed after the depletion of the other SAC proteins (Fig 5A and 5B). Time-lapse microscopy analysis of cells stably expressing H2B-GFP and mCherry-Tubulin confirmed that mitotic timing was significantly shorter in Zw10-depleted cells than in control cells (Fig 6D and 6E; S9 and S10 Videos), which fits to the Premature Sister Chromatids Separation (PSCS) phenotype described in Zw10 mutant embryos and larval tissues [43, 51, 52]. And indeed, Zw10 depletion led to a 30% reduction of newly synthesized SNAP-CENP-A (Fig 6F and 6G) and SNAP-CAL1 (S7A and S7B Fig) at centromeres compared to control-depleted cells.

To exclude a potential role of Zw10 in CENP-A stability we performed a SNAP-tag pulse- chase experiment in Zw10-depleted cells (S7C and S7D Fig) in which we labeled centromeric SNAP-CENP-A at t0 and measured its remaining levels after 24 hours. This showed that centromeric SNAP-CENP-A was diluted 2-fold after 24 hours as expected after 1 cell cycle both in control and Zw10-depleted cells (S7C and S7D Fig). Furthermore, FRAP analysis revealed that the recovery rate of GFP-CENP-A at centromeres was comparable in control and Zw10-depleted cells (S7E and S7F Fig). Thus, the CENP-A mitotic loading machinery is functional in Zw10-depleted cells suggesting that Zw10 does not influence CENP-A loading directly but possibly through its role in checkpoint activity.

### Zw10 mediates the recruitment of the RZZ to the kinetochore independently of Spc105R

**Fig 7:**
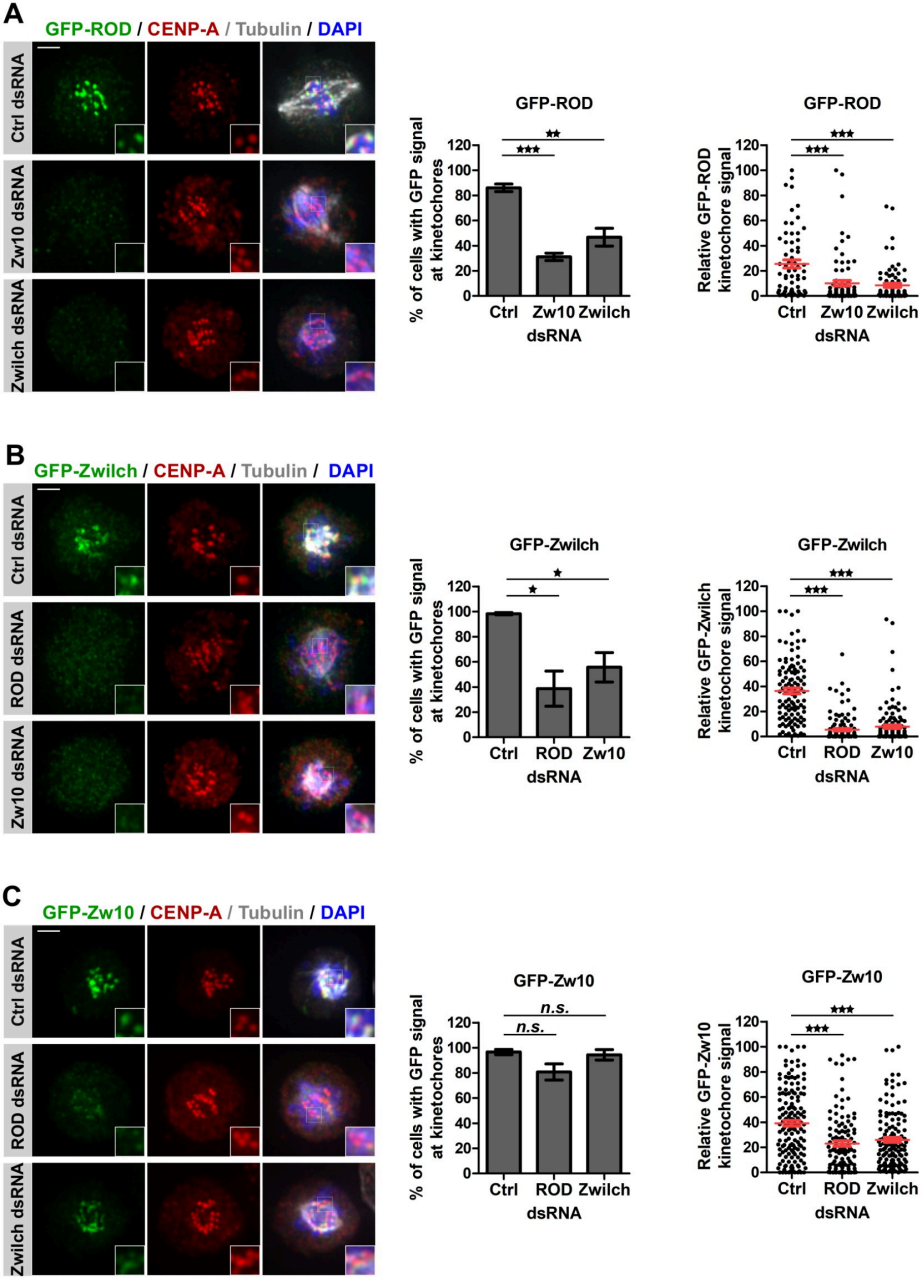
Zw10 is the most proximal RZZ component at kinetochores in Drosophila. **A. Left panel** Immunofluorescence of cells expressing GFP-ROD and mCherry-Tubulin after 96 h depletion of RZZ components stained with anti-GFP antibody (green) and anti-CENP-A (red), DNA (DAPI) is shown in blue. ***Middle panel***. Prometaphase cells were counted for the presence or absence of GFP signal at kinetochores. ***Right panel***. Quantification of total GFP-ROD kinetochore intensity per cell. GFP fluorescence intensity at kinetochores was measured for each cell and normalized within one experiment before pooling measurements from at least 3 experiments per condition. **B**. Similar experiments as in A were performed in GFP-Zwilch expressing cells. **C**. Similar experiments as in A were performed in GFP-Zw10 expressing cells. Mean +/- SEM, n>100 cells. Student’s t-test (*n*.*s*.: non-significant; *: p<0.05; **: p<0.01***: p<0.001). Scale bar: 2 μm.

**Fig 8:**
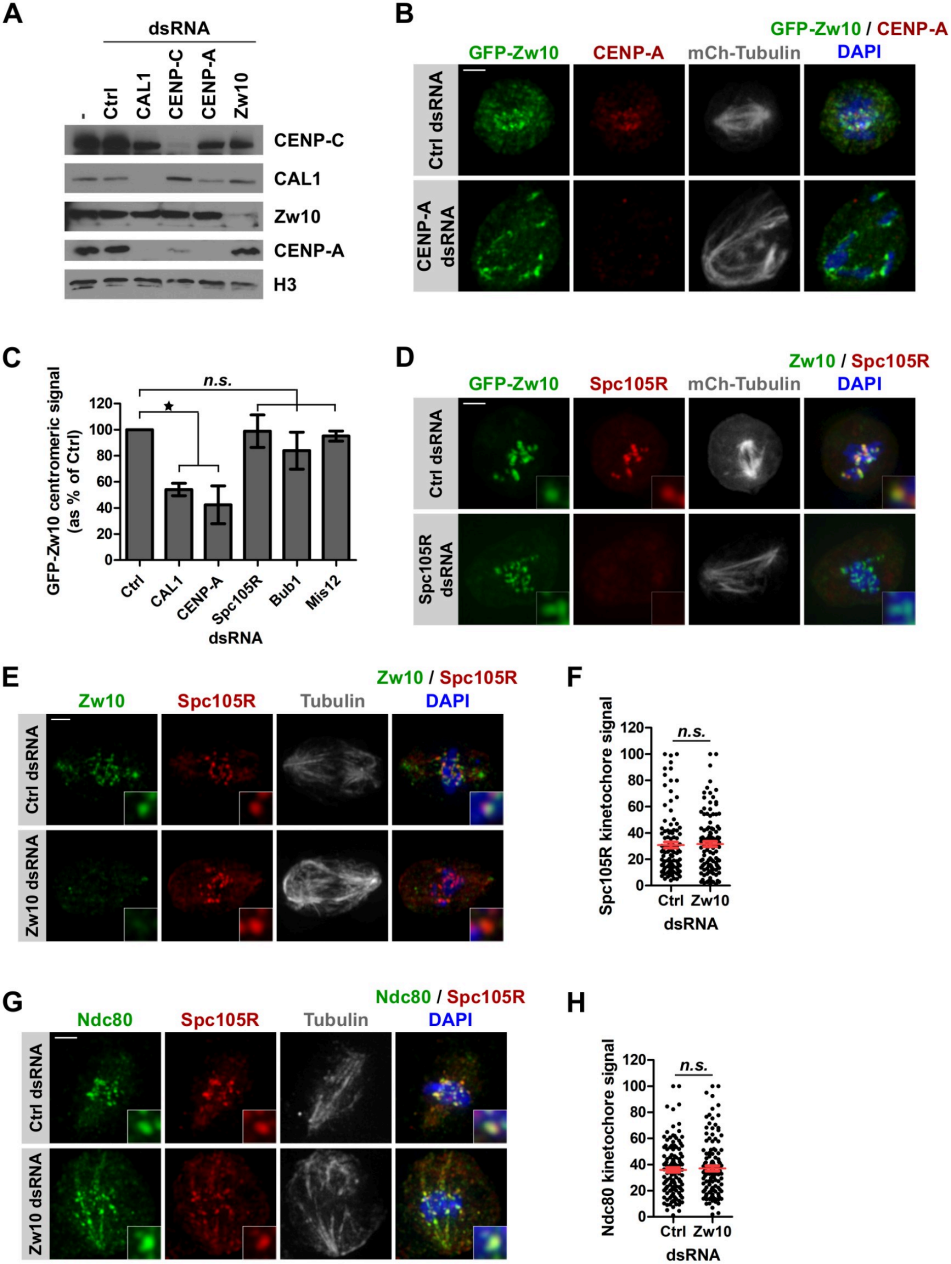
RZZ recruitment to the kinetochore depends on direct interaction with inner centromere proteins. **A**. Immunoblot using CENP-C, CAL1, Zw10 and CENP-A antibodies showing protein levels in S2 cells after 96 h dsRNA treatment as indicated. H3 serves as a loading control. **B**. Immunofluorescence of GFP-Zw10/mCherry-Tubulin expressing cells with anti-GFP (green), anti-CENP-A (red) antibodies after 96 h depletion of CENP-A. DNA (DAPI) is shown in blue. Scale bar: 2 μm. **C**. Quantifications showing the total GFP-Zw10 centromeric intensity per nucleus as % of control in the indicated dsRNA-treated cells. Prometaphase cells were selected for analysis. Mean +/- SEM of 3 experiments (n>100 cells), Student’s t-test (*n*.*s*.: non-significant; *: p<0.05). **D**. Immunofluorescence of GFP-Zw10/mCherry-Tubulin expressing cells with anti-GFP (green) and anti-Spc105R antibodies (red) after 96 h Spc105R depletion. DNA (DAPI) is shown in blue. **E**. Immunofluorescence of S2 cells with anti-Zw10 (green), anti-Spc105R (red) and anti-tubulin (grey) antibodies after 96 h of Zw10 depletion, DNA (DAPI) is shown in blue. Scale bar: 2 μm. **F**. Quantification of E showing the total Spc105R kinetochore intensity per cell. Spc105R fluorescence intensity at kinetochores was measured for each cell and normalized within one experiment before pooling measurements from at least 3 experiments per condition. Mean +/- SEM (n>90 cells), Student’s t-test (n.s.: non-significant) **G**. Immunofluorescence of S2 cells with anti-Ndc80 (green), anti-Spc105R (red) and anti-tubulin (grey) antibodies after 96 h Zw10 depletion, DNA (DAPI) is shown in blue. Scale bar: 2 μm. **H**. Quantification of G showing the total Ndc80 kinetochore intensity per cell. Ndc80 fluorescence intensity at kinetochores was measured for each cell and normalized within one experiment before pooling measurements from at least 3 experiments per condition. Mean +/- SEM (n>90 cells), Student’s t-test (*n*.*s*.: non-significant).

How RZZ is recruited to the kinetochore at mitosis onset remains elusive in Drosophila. We first sought to determine if one of the RZZ components is responsible for targeting the whole complex to the kinetochore. We established stable cell lines expressing GFP-Zwilch and GFP-ROD and confirmed that the localization of the tagged proteins is consistent with previous reports [46, 51, 53, 54] (S6E and S6F Fig). Knockdown of RZZ complex components (S8A Fig) led to the expected spindle morphology defects in all cell lines (Fig 7) [43, 54–56]. Importantly, depletion of any complex component led to a reduction of the other GFP-RZZ proteins at kinetochores (Fig 7) suggesting that the RZZ components are mutually dependent on each other similar to what has been reported in Drosophila tissues [42, 44, 54, 56, 57]. However, levels of GFP-Zw10 at kinetochores were the least affected and the reduction was statistically non-significant indicating that a substantial amount of GFP-Zw10 is still recruited to kinetochores in the absence of its complex partners ROD or Zwilch (Fig 7C). This suggests that Zw10 is the most proximal component of the complex at kinetochores in Drosophila cells. Like all kinetochore proteins tested so far [58], the localization of Zw10 to the kinetochore is abolished when CENP-A (Fig 8B and 8C) or its loading factor CAL1 (Fig 8C and S8B Fig) are depleted, even though total Zw10 protein levels remained largely unchanged (Fig 8A).

In mammalian cells, several pathways have been implicated in the recruitment of the RZZ complex including KNL1, Bub1, and Zwint [59–63]. As no ortholog of Zwint has been identified in Drosophila so far, we tested if the Drosophila KNL1 ortholog Spc105R or Bub1 are required for RZZ recruitment to the kinetochore. Neither depletion of Spc105R nor Bub1 (S8A Fig) affected Zw10 localization (Fig 8C and 8D and S8C Fig). We also tested Mis12, which has been shown to localize to kinetochores throughout the cell cycle [58] and found that it is also not involved in Zw10 recruitment to the kinetochore (Fig 8C and S8A and S8D Fig). We also excluded the KMN network since the recruitment of the KMN network depends on the prior localization of Spc105R and Mis12 at kinetochores [58]. Conversely, depletion of Zw10 (Fig 8A) did not prevent Spc105R (Fig 8E and 8F), Mis12 (S8E and S8F Fig), Ndc80 (Fig 8G and 8H) or BubR1 (S8G and S8H Fig) localization to the kinetochore. Therefore, we propose that the Drosophila checkpoint proteins and outer kinetochore components are assembled through two independent branches (S8I Fig): on the one hand, CAL1 directly recruits the RZZ complex, which is necessary to localize the MAD proteins to the kinetochore [46, 50], and on the other hand, CENP-C attracts Mis12-Spc105R, which are required to recruit all other components of the KMN network as well as the Bub proteins [28, 58, 64–66]. We conclude from these results that there is a direct connection of the SAC and the loading of CENP-A via CAL1 and we speculate that the CAL1-RZZ branch might regulate CENP-A loading and mitotic duration, thereby controlling CENP-A loading in a cell cycle time-dependent manner.

## Discussion

In Drosophila cells, CENP-A loading takes place primarily during prometaphase-metaphase [10]. Additional turnover of CENP-A in G1 has been reported leading to the hypothesis that CENP-A could be further incorporated at this stage [12], which we did not observe in our FRAP experiments when we bleached centromeric CENP-A at the end of cytokinesis. However, our FRAP experiments and most importantly our live staining of newly synthesized SNAP-CENP-A confirmed the observations reported by Mellone et al., that the majority of CENP-A loading takes place during mitosis in Drosophila cultured cells [10].

**Fig 9:**
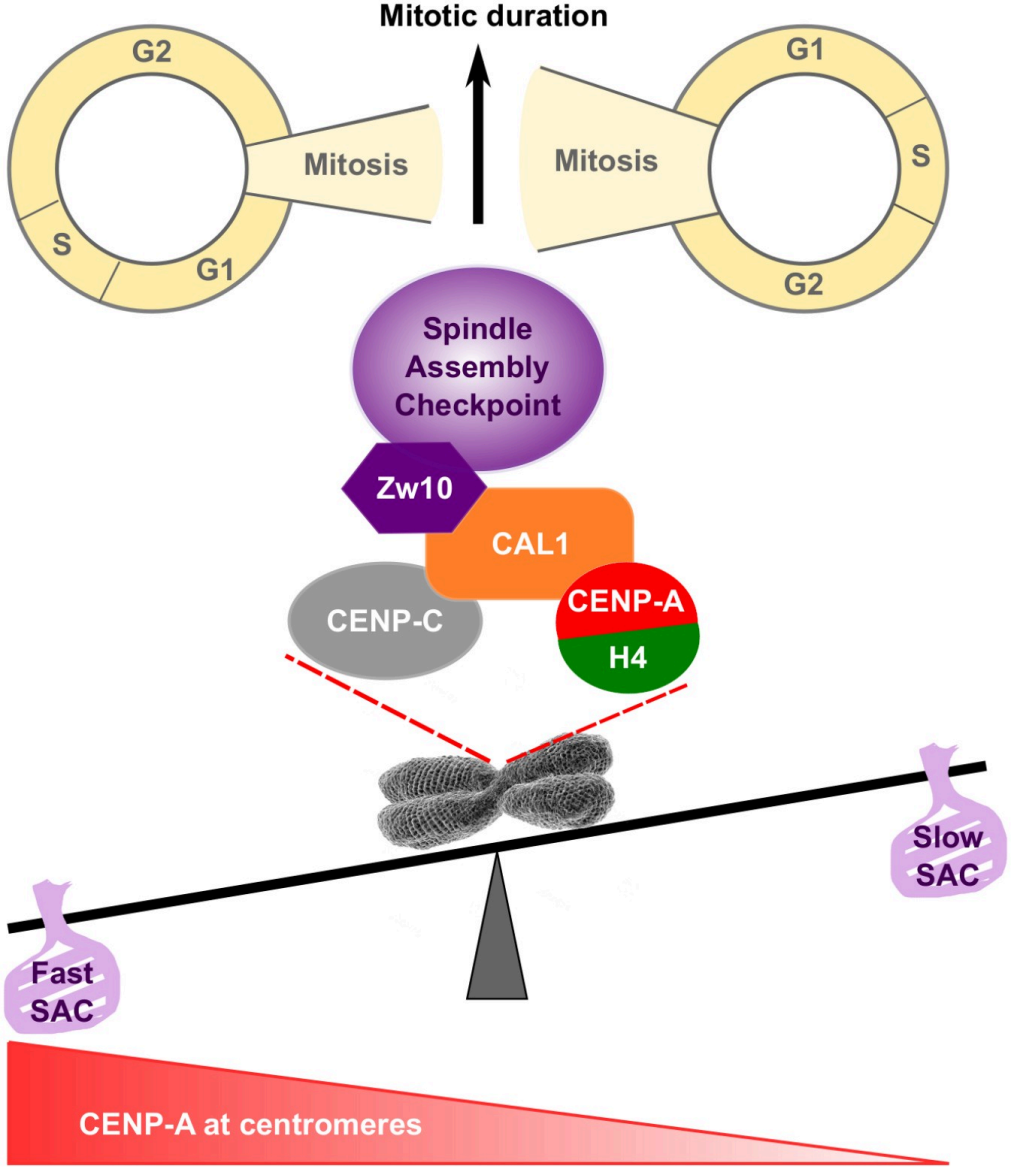
Model. The model depicts the relationship between the amount of CENP-A at centromeric chromatin at the onset of mitosis and the duration of mitosis. Cells that have a relative large pool of CENP-A at centromeres go through mitosis faster. The precise reason for this decreased mitotic timing is still unknown but we hypothesize that cells with sufficient CENP-A recruit SAC components more quickly or capture microtubules more efficiently to satisfy the SAC faster. This hypothesis is supported by our finding that the CENP-A loading factor CAL1 directly interact with the SAC component and RZZ subunit Zw10. More CAL1 may be able to not only recruit more CENP-A by priming centromeres and loading more efficiently CENP-A but also may recruit the RZZ complex and the SAC more efficiently. Vice versa, an active SAC may give the cell more time to recruit sufficient CENP-A via CAL1 to ensure that the cell does not progress further without having loaded sufficient CENP-A to the centromere.

In flies, CENP-A incorporation is controlled by its chaperone CAL1 [17, 19, 30]. It has been shown previously that co-overexpression of exogenous CENP-A and CAL1 leads to an increase of centromeric CENP-A in embryos [30]. We now show that overexpression of CAL1 alone leads to increased endogenous CENP-A protein levels in Drosophila cultured cells. Ectopic incorporation of CENP-A, however, was never observed suggesting that CAL1 loads CENP-A exclusively to centromeres and that ectopic CENP-A incorporation in flies depends on alternative loading mechanisms similar to what has been suggested in human cells [67]. Importantly, increased centromeric CENP-A levels following CAL1 overexpression correlated with faster mitosis. A similar acceleration of mitotic timing was observed when CENP-A was only mildly overexpressed, revealing a possible link between CENP-A loading and mitotic timing. Indeed, shortening mitosis duration by depleting Mad2 or BubR1 [68] was associated with decreased CENP-A loading. However, just elongating the mitotic time window during which CENP-A can get loaded (Spindly or Cdc27 depletion, or by drug treatment) did not increase the amount of CENP-A incorporated at centromeres, showing that the length of mitosis alone is insufficient to control CENP-A amounts at centromeres. Rather, these experiments showed that only a defined amount of CENP-A can be incorporated at each mitosis probably correlating with CAL1’s availability. Indeed, live analysis of CAL1-overexpressing cells allowed us to visualize newly synthesized CENP-A incorporation to centromeres in all stages of the cell cycle. This strongly suggests that CAL1 controls CENP-A incorporation into centromeric chromatin both quantitatively and temporally. How exactly CENP-A levels at centromeres are sensed is unclear but we identified the RZZ-component Zw10 as a new CAL1 interacting partner, which directly connects CENP-A loading to the SAC. It has been proposed that SAC activation is a 2-steps process [35]: at the end of G2-beginning of mitosis, before the kinetochores are assembled, cytosolic Mad1-Mad2 dimers initiate MCC formation, which inhibit APC/C^Cdc20^ and determine the timing of mitosis [37, 38, 68–71]. After nuclear envelope disassembly, kinetochore-dependent MCC are generated and regulated by kinetochore-microtubules attachment. Therefore, we suggest the following model (Fig 9): efficient CENP-A loading by CAL1 during mitosis recruits Zw10 up to a threshold, which is sensed by the SAC. Low CENP-A levels at centromeres could lead to more cytosolic Mad2 thereby keeping the timer active longer [35]. Higher CENP-A levels at centromeres during early mitosis would accelerate the recruitment of RZZ and consequently Mad2 to the kinetochores or capture microtubules more efficiently, therefore, releasing the timer and shortening mitosis duration in cells where kinetochores attach properly to the spindle microtubules. Interestingly, Nocodazole treatment did not affect CENP-A loading confirming previous observations that kinetochores attachment to the microtubule spindle does not play a role in CENP-A loading [9, 10]. These results are pointing further to an additional function of the SAC independent of the control of microtubule attachment. Interestingly, recent evidence shows that RZZ together with Spindly plays a central role in kinetochore expansion during early mitosis to form a fibrous corona that then compacts upon microtubule capture [72–74]. Whether and -if so- how the kinetochore expansion by RZZ and spindly is involved in CENP-A loading needs to be investigated in the future.

Many essential components of the SAC require outer kinetochore components for their localization to centromeric regions. However, several outer components are missing from the Drosophila kinetochore [58, 75] and even though Mad1/2 recruitment to the kinetochore depends on the RZZ complex [50], the factors necessary for the localization of the RZZ to kinetochores are unknown. We showed here that RZZ localization to the kinetochores does not require KNL1^Spc105R^ but depends on the centromeric proteins CAL1 and CENP-A. Therefore, we propose that the Drosophila outer kinetochore and components of the SAC assemble through two independent pathways (S8I Fig): the CENP-C-KMN-Bub1-Bub3/BubR1 branch [28, 64, 66] or the CAL1-RZZ-Mad1/2 branch. How those two pathways communicate for the formation of MCC complexes remains to be determined. One link may be the KMN complex since Mad2 is diminished in the absence of KMN proteins [39]. Interestingly, Spc105R mutation does not affect SAC function in fly embryos suggesting that flies rely more on the RZZ-Mad1/2 branch to engage the SAC [64].

CENP-A expression [76] and its stability [22, 29] together with its dependence on the low abundant and highly specific loading factor CAL1 and the here shown connection to mitotic events are likely interconnected cellular surveillance mechanisms to avoid misincorporation of CENP-A and, therefore, securing genome stability. How CAL1 itself is regulated to obtain such specificity is currently unknown. In conclusion, we show that there is direct crosstalk between the SAC and the maintenance of centromeric chromatin, ensuring mitotic fidelity not only by controlling microtubule attachment but also by regulating the accurate composition of centromeres.

## Materials and methods

### Gene constructs

The plasmid encoding CENP-A tagged with an N-terminal EGFP has been described previously [19] as well as those encoding CENP-A and CAL1 with N-terminal SNAP-tag [10]. Constructs created for this study (GFP-ROD, GFP-Zwilch, GFP-Zw10, GFP-Mad2) were cloned into AscI and PacI sites of the pCopia-localization and purification (LAP) vector with a basal expression Copia promoter and an N-terminal mEGFP tag [19]. For overexpression studies, CAL1-V5 and CENP-A-GFP were cloned into a pMT vector (Invitrogen). For the yeast two-hybrid studies, pMM6 plasmids containing full-length CAL1, CAL1 truncations, and CENP-A have been described in [29]. Bub1, Bub3, BubR1, Mad1, Mad2, Zw10 (full-length, aa1-240, aa241-481, aa482-721), Zwilch, and ROD (full length, aa1-696, aa697-1392, aa1393-2090) were cloned into pMM5 plasmids. For bacterial expression, pCA528 plasmids containing CAL1 and CENP-A have been described in [29]. Zw10 was cloned into pGEX5x-1. Following stable cell lines with either a constitutive copia promoter or a CuSO_4_-inducible Metallothionein promoter were created: pCopia-SNAP-CENP-A (98% transfection efficiency, TE) used in Figs 3A, 5A, 5B, 6F and 6G, S5B–S5E, S7C and S7D Figs; mCherry-Tubulin (TE not determined, ND) pCopia-EGFP-CENP-A (92% TE) used in Figs 3A, 4 and 5C, S1A, S5A, S7E and S7F Figs; mCherry-Tubulin (TE ND) pMT-CENP-A-GFP (93% TE) used in Fig 3, S4 Fig; H2B-GFP (TE ND) mCherry-Tubulin (TE ND) pMT-CAL1-V5 (54% TE) used in Fig 2F and 2G, S2C and S2D Fig; mCherry-Tubulin (TE ND) pMT-CENP-A-GFP (93% TE) pMT-CAL1-V5 (83% TE) used in Figs 1E, 1G, 1H, 2A and 2B, S2A, S2B, S2E, S2F, S2H, S3A, S3C–S3E Figs; mCherry-Tubulin (TE ND) pCopia-SNAP-CENP-A (95% TE) pMT-CAL1-V5 (79% TE) used in Figs 1E, 1I, 1J, 2C–2E, S1B–S1E Fig; pCopia-SNAP-CAL1 (95% TE) used in S5F, S7A and S7B Fig.

### Cell culture

#### Cell maintenance

Drosophila S2 cells were maintained under sterile conditions at 25°C in Schneider medium containing 10% heat-inactivated fetal bovine serum (FBS) and 100 μg/ml Penicillin/Streptomycin. Expression of genes under the metallothionein promoter (pMT vector) was induced by supplementing the medium with CuSO_4_ (CENP-A-GFP: 10 μM for 2 h; CAL1-V5: 100 μM for 24 h).

#### RNAi and transfection experiments

Double-stranded RNA (dsRNA) was produced using the MEGAscript (Ambion) kit according to the manufacturer’s protocol. RNAi was carried out as described previously [19]. In short, 10^6^ S2 cells were incubated for 1 h with 15 μg dsRNA in serum-free medium. Subsequently, 2 volumes of 15% serum-containing medium were added. The analysis was usually performed 96 h after treatment.

Stable cell lines were generated by co-transfecting the plasmid of interest together with a plasmid carrying the resistance gene for hygromycin with Cellfectin II (Life Technologies), and the selection process was started 2 d after transfection by supplementing the media with 250 μg/ml Hygromycin B.

#### SNAP quench chase pulse experiments

Cells expressing either SNAP-tagged CENP-A or CAL1 proteins were treated with dsRNA as described in the RNAi section or incubated with CuSO_4_ to induce pMT-CAL1-V5 expression. Cells were then collected and incubated in fresh SM containing 10 μM SNAP-Cell Block (New England Biolabs S9106), for 30 min at 25°C, 400 rpm. Cells were then pelleted, washed 3 times (1^st^ wash for 30 min, at 25°C, 400 rpm) and plated in conditional medium. After 24 h, cells were collected and incubated with 4 μM SNAP-Cell TMR-Star (New England Biolabs S9105) or SNAP-SiR647 (New England Biolabs S9102) for 15 min at 25°C, 400 rpm, protected from light. The cells were then pelleted, washed 3 times with SM (1^st^ wash for 30 min, at 25°C, 400 rpm), one time in PBS and prepared for imaging. Cells were settled on a glass coverslip for 10 min, fixed with 4% PFA for 10 min, washed three times in PBS, and counterstained with DAPI before mounting.

#### SNAP pulse-chase

Cells expressing SNAP-tagged CENP-A proteins were treated with dsRNA as described in the RNAi section. After 3 days, cells were collected and incubated in fresh medium containing 4 μM SNAP-Cell TMR-Star (New England Biolabs, Inc.) for 15 min at 25°C, 400 rpm, protected from light. Cells were then washed 3 times with SM (1^st^ wash for 30 min, at 25°C, 400 rpm) and plated in conditional medium. After 24 h, cells were collected and prepared for imaging as previously described.

#### SNAP live imaging

To follow the incorporation of newly synthesized SNAP-CENP-A, SNAP-CENP-A was blocked as previously described (see section SNAP quench chase pulse). Cells were then resuspended in conditional medium containing 0.5 μM SNAP-640 dye [32] and placed into an Ibidi imaging chamber before imaging.

### Immunofluorescence (IF)

IF was essentially performed as described previously [19]. Cells were harvested, washed once with PBS and placed to settle on a glass coverslip for 10 min before fixation with 4% PFA for 10 min. Cells were washed three times in PBS and permeabilized for 5 min with PBS containing 0.5% Triton X-100. Unspecific binding was prevented by blocking the cells for 30 min with 1% BSA in PBS. Primary antibodies diluted in blocking solution were incubated for 30 min at RT. After three washes with PBS, cells were incubated with the corresponding, fluorescently labeled secondary antibodies (diluted in blocking solution) for 30 min at RT. The secondary antibody incubation and all the following steps were performed while protected from light. After three washes in PBS, DNA was stained for 5 min with DAPI (1 μg/ml in PBS). The cells were then washed two more times with PBS before mounting in Aqua/Polymount medium (Polysciences, Inc.) on a glass slide.

### Preparation of mitotic chromosome spreads

Mitotic chromosome spreads were essentially performed as described previously [77]. To obtain mitotic chromosomes, 2x10^5^ exponentially growing cells were arrested in mitosis with 2.5 μg/μl Colcemid for 1 h, centrifuged for 3 min at 800 *g*, resuspended in 0.5 ml hypotonic sodium citrate solution (0.5% Na-citrate in ddH2O), and incubated for 8–10 min. Cells were spun on positively charged slides in a cytocentrifuge (Shandon 4 Cytospin; Thermo Fisher Scientific) at 900 rpm for 10 min. Slides were placed 10 min in KCM buffer (120 mM KCl, 20 mM NaCl, 10 mM Tris-HCl ph7.7, 0.1% Triton X-100) before 30 min incubation with primary antibodies diluted in TEEN buffer (1 mM Triethanolamine: HCl pH8.5, 0.2 mM EDTA, 25 mM NaCl, 0.1% Triton X-100, 0.1% BSA) at 37°C. After 3 washes in KB buffer (10 mM Tris-HCl pH7.7, 0.15 M NaCl, 0.1% BSA) at RT, slides were incubated with secondary antibodies diluted in TEEN buffer for 30 min at 37°C. Slides were then washed 3 times in KB before incubation with 1 μg/ml DAPI diluted in PBS for 5 min at RT. Slides were then mounted in Aqua/Polymount medium (Polysciences, Inc.).

### Kinetochore-microtubule interaction assay (MG132-Taxol assay)

After induction (24 h for CAL1-V5-overexpressing cells, 2 h for CENP-A-GFP-overexpressing cells), cells were washed and incubated with 20 μM MG132 for 1 h. Cells were then washed before incubation with 100 nM Taxol in conditional medium for 3 h. Cells were then fixed and stained with DAPI. Cells were scored into 2 categories: either all kinetochores are attached to microtubules (attached) or at least 1 kinetochore lacks microtubule attachment (>1 unattached).

### Microscopy and image analysis

All images were acquired on a DeltaVision Core system (Applied Precision) equipped with an Olympus UPlanSApo 100X oil immersion objective (n.a. 1.4) at binning 1x1 (mitotic figures) or 2x2 (interphase). Images were taken as z stacks of 0.3 μm increments to the exception of the kinetochore-microtubule assay where 0.2 μm z stacks were used. All images were deconvolved (20 cycles additive with noise filtering) and maximum-projected using the Applied Precisions soft-WoRx 3.7.1 suite. Image quantification was done using ImageJ software. For determining the number and intensities of centromere/kinetochore dots, a set of plugins developed by the Nikon Imaging Centre, University Heidelberg was used. After marking the nuclear boundaries in the DAPI channel and saving them with the ROI tool, the spots in the channel of interest were enhanced using the DoG spot enhancer plugin. The threshold was adjusted using ‘‘Li” instead of default settings. The number of spots with their mean intensities relative to the nuclear area and the total particle intensity per nucleus were determined using the ROI particle analyzer plugin.

Time-lapse imaging was performed on a DeltaVision Core system (Applied Precision) equipped either with an Olympus 60X/1.42, N Plan Apo oil immersion objective or an Olympus 40X/1.35, UApo/340 oil objective, at binning 2x2 with 3 min (mitosis duration and fidelity measurements) or 15 min (SNAP-CENP-A live imaging) time-lapse series, for 16 h, with z stacks of 1 μm increment.

FRAP was performed on a Zeiss LSM 780 confocal microscope equipped with a 63x/1.4 oil immersion objective. The number of focal planes per time point, as well as time intervals and total duration, were adjusted according to experimental needs. The spacing of focal planes was 1 μm in all experiments. Recovery of the EGFP-CENP-A signal was analyzed using FRAP analysis plugin developed by ZMBH imaging facility. After marking the boundaries of the cell of interest, the threshold allowing visualization of centromeric signal exclusively was defined on a pre-bleach image. The total intensity of the thresholded signal was then measured for each time point and area. Similar measurements were performed on non-bleached cells from the same area to determine the photobleaching level in each time-series. After correction, EGFP-CENP-A signal intensity was plotted against the time, a linear regression curve was fitted.

### Statistics

Microscopy data were quantified first separately and then averaged to facilitate the comparison of different treatments and knockdown. For each experiment, the ratio of the sample mean to the control mean was calculated and converted to percentage with the control condition set to 100%. The ratios derived from at least 3 independent experiments were then averaged and are presented on graph bars. When dot plots are presented, at least 3 independent experiments were analyzed and normalized using the highest value obtained as 100. This allowed the pooling of different experiments and the representation of each analyzed cell as dot.

### Protein purification and GST-pulldown

The Zw10 coding sequence was cloned into a pGEX5x-1 bacterial expression vector and transformed into BL21DE3 bacteria. Expression was induced by addition of 1 mM IPTG at OD = 0.6. After 16 h at 21°C, bacteria were pelleted and resuspended in lysis buffer (1% Triton X-100, 1 mM DTT, 1 mg/ml Lysozyme, 2 mg/ml aprotinin, 5 mg/ml leupeptin, 1 mg/ml pepstatin in PBS). After 30 min incubation at 4°C, bacteria were sonicated (10 cycles, 30 seconds pulse-30 seconds pause, 60% output power). The lysate was then centrifuged at 15000 g for 45 min. The supernatant was then incubated with Protino glutathione-agarose 4B (Macherey-Nagel) for 1 h at 4°C. After 6 washes with 1% Triton X-100 in PBS, GST-Zw10 was eluted with 20 mM reduced glutathione, 50 mM Tris-HCl pH 8, 1 mM DTT. Buffer was then exchanged using Amicon Ultra 50 K with storage buffer (50 mM Tris-HCl pH 8, 1 mM DTT). His-Sumo-CAL1 and His-Sumo-CENP-A purifications have been described in [29].

Pulldown assays were performed by incubating GST or GST-Zw10 coupled beads with 100 ng His-Sumo-CAL1 or His-Sumo-CENP-A in interaction buffer (20 mM Tris pH 8, 150 mM NaCl, 0.5 mM EDTA, 10% glycerol, 0.1% NP-40, 1 mM DTT) for 1 h at 4°C. Beads were then washed 6 times in interaction buffer before resuspension in 1 bead volume 2X sample Laemmli buffer and boiling at 95°C for 5 min.

### Immunoprecipitation

GFP-tagged proteins were isolated using a GFP-specific single-chain antibody (GBP) coupled to NHS-activated Sepharose (GE) [78]. A total of 1.0–2.0 x 10^8^ cells were lysed in two pellet volumes of 20 mM Tris-HCl pH 7.5, 1% NP-40, 250 mM NaCl, 20 mM NEM, 10 mM NaF, 2 mM PMSF, 2 mg/ml aprotinin, 5 mg/ml leupeptin, 1 mg/ml pepstatin for 15 min at 4 °C. After clearing the lysate by centrifugation, the supernatant was incubated for 2 h at 4 °C with 50 μl pre-equilibrated GBP-beads. The beads were washed six times with lysis buffer and eluted with 2x Laemmli buffer 5 min at 95°C.

### WB analysis and quantification

Cell lysates were separated on a 4–15% SDS poly-acrylamide gel, transferred onto a nitrocellulose membrane for 2 h at 100 V (Tris-Glycin buffer containing 0.1% SDS). After blocking in 5% milk in PBST, primary antibodies were incubated O/N at 4°C in the blocking solution. After washing, secondary antibodies coupled to horseradish peroxidase were added for 1 h at RT before ECL detection (Thermo Fisher Scientific). Immunoblots were quantified using ImageJ.

### Antibodies

The following primary antibodies were used: rabbit H3 (Abcam AB 1791), rabbit CAL1 [29], chicken CENP-A (commercially obtained from Dr. Heun), rabbit CENP-A (Active Motif 39713), rabbit YFP (from Pr. Bukau), mouse tubulin (Sigma T9026), mouse V5 (Invitrogen V8012), sheep Scp105R (from Dr. Glover), rabbit Zw10 (from Dr. Goldberg), guinea pig CENP-C [19], rabbit phospho-S10 histone H3 (Abcam ab5176), BubR1 (from Dr. Glover), goat GST (GE healthcare 27457701), rabbit His (Abcam ab9108), rabbit Ndc80 [79], rabbit Mis12 [79], rabbit Mad2 (from Dr. Sunkel). Secondary antibodies coupled to Alexa Fluor 488, Alexa Fluor 546 and Alexa Fluor 647 fluorophores (Molecular Probes) were used for IF, and horseradish peroxidase-conjugated secondary antibodies (Sigma) were used for WB analysis.

### Reverse transcription and quantitative qPCR

RNA was isolated using Trizol(R) according to the manufacturer’s procedures. Whole transcriptome cDNA synthesis was performed using the Quantitect Reverse Transcription kit (Invitrogen), with a combination of oligo (dT) and random hexamer primers in equal proportions. A control reaction with no reverse transcription was always performed in parallel.

qPCR was performed after cDNA synthesis on a LightCycler 480 (Roche) using LightCycler 480 SYBR Green I Master (Roche). All reactions were run in triplicate in a LightCycler 480 multiwell plate. Actin was used as a reference. The level of each targeted gene in the control mock-treated sample was normalized to 1, and compared with the corresponding–depleted samples.

### Yeast two-hybrid

Protein-protein interactions were tested using pMM5 and pMM6 fusion constructs and the yeast strain SGY37VIII. The YTH was performed as described previously [80]. In short, interactions were judged based on the activity of β-galactosidase that results in the conversion of X-Gal (5-bromo-4-chloro-3-indolyl ß-D-galactopyranoside) into a blue dye.

## Supporting information

S1 Fig Related to Figs 1 and 2. **A**. FRAP of GFP-CENP-A in G1 phase. Cells expressing GFP-CENP-A and mCherry-Tubulin were followed through mitosis, GFP-CENP-A signal was partially bleached in early G1 and cells were further imaged for > 2 h. Time-lapse: 6 min. Scale bar: 2 μm. The total GFP-CENP-A centromeric signal of 6 cells is shown as mean +/- SEM. **B**. SNAP Quench-Chase-Pulse experiment in MG132-treated cells. After 24 h induction of pMT-CAL1-V5, cells were incubated with MG132 for 2 h to arrest cells in mitosis prior to the SNAP-block. A 4-h chase was performed in presence of MG132 to allow synthesis and incorporation of new SNAP-CENP-A into centromeres before staining with SNAP-Si647 and fixation. The graph shows the percentage of cells with SNAP-CENP-A at centromeres. **C**. Immunofluorescence of SNAP-CENP-A in control (non-induced) or induced (24 h) pMT-CAL1-V5 cells. The cells were incubated with SNAP-Block, washed, incubated with SNAP-640 dye for 20 h before immunostaining with an anti-CENP-A antibody (green), or taken directly after block (0 h), and stained with SNAP-640 dye for 15 min to check the efficiency of the block. DNA (DAPI) is shown in grey. Scale bar: 2 μm. **D**. Quantification of C showing the percentage of cells positive for centromeric SNAP-CENP-A staining. **E**. Quantification of C showing the total SNAP-CENP-A centromeric intensity per nucleus as % of control. All graphs show Mean +/- SEM of 3 experiments (n>300 cells), Student’s t-test (n.s.: non-significant; *: p<0.05; **: p<0.01).
(TIF)

S2 Fig Related to Fig 2. **A**. Quantification showing GFP-CENP-A centromeric signal intensity per nucleus at t0 of time-lapse imaging with or without pMT-CAL1-V5 induction. Mean +/- SEM, n>80 cells. Student’s t-test (***: p<0.001). Data from 2 experiments were normalized and combined. **B**. Time-lapse imaging of GFP-CENP-A/mCherry-tubulin expressing cells with or without prior pMT-CAL1-V5 induction (100 μM CuSO_4_, 24 h). Imaging: 16 h. Time-lapse: 3 min. Scale bar: 2 μm. **C-D** Mitotic phenotypes of CAL1 overexpression. pMT-CAL1-V5 expression was induced for 24 h in H2B-GFP/mCherry-Tubulin cells. Cells were imaged for 16 h and scored for the accuracy of mitosis: lagging (presence of lagging chromosomes during anaphase that will resolve before cytokinesis)(**C**) or defective (formation of tripolar spindles, multinucleated cells)(**D**). Mean +/- SEM n > 200 cells. Student’s t-test (*n*.*s*.: non-significant). **E-G**. Amount of kinetochore proteins recruited during mitosis in the presence or absence of CAL1 overexpression. pMT-CAL1-V5 expression was induced for 24 h in GFP-CENP-A/mCherry-Tubulin cells (**E-F**) or GFP-Zw10 expressing cells (**G**). Fixed cells were stained with anti-CENP-C (**E**, red), anti-Spc105R (**F**, red) or anti-tubulin (**G**, grey). DNA (DAPI) is shown in blue. Scale bar: 2 μm. Kinetochore signal intensity of the indicated proteins is shown as % of control. Only prometaphase cells were analyzed. Mean +/- SEM of 3 experiments (n > 90 cells). Student’s t-test (*n*.*s*.: non-significant). **H**. Kinetochore-microtubule attachment assay. After 24 h pMT-CAL1-V5 induction, GFP-CENP-A/mCherry-Tubulin cells were incubated with 20 μM MG132 for 1 h, then with 100 nM Taxol for 3 h. The fixed cells were scored into 2 categories: either “attached” when each kinetochore was attached to microtubules, or “> 1 unattached” when at least 1 kinetochore was not stably connected to microtubules. The graph shows the percentage of cells in each category as Mean +/- SEM, N = 2.
(TIF)

S3 Fig Related to Fig 2. **A-B**. Amount of SAC proteins recruited to the kinetochore during mitosis in the presence or absence of CAL1 overexpression. pMT-CAL1-V5 expression was induced for 24 h in GFP-CENP-A/mCherry-Tubulin cells (**A**) or GFP-Mad2/mCherry-Tubulin (**B**) expressing cells. Fixed cells were stained with anti-BubR1 (**A**, red). DNA (DAPI) is shown in blue. Scale bar: 2 μm. Kinetochore signal intensity of the indicated proteins is shown as % of control. Only prometaphase cells were analyzed. Mean +/- SEM of 3 experiments (n > 90 cells). Student’s t-test (*n*.*s*.: non-significant). **C**. Stills from time-lapse imaging experiments showing GFP-CENP-A/mCherry-Tubulin cells with or without prior pMT-CAL1-V5 induction (24 h) imaged immediately after the addition of 1 μM Taxol and after 4 h. Imaging: 16 h. Scale bar: 2 μm. **D**. Quantification of C showing the percentage of cells that arrest (‘arrest’) in response to Taxol treatment or arrest and restart to finish mitosis (‘finish’). **E**. Quantification of time-lapse imaging showing the time taxol-treated pMT-CAL1-V5-overexpressing cells spend in an arrested state before either reverting to G2-like state or proceeding through mitosis. Mean +/- SEM, n>100 cells. Student’s t-test (***: p<0.001).
(TIF)

S4 Fig Related to Fig 3. **A**. CAL1 centromeric levels in pMT-CENP-A-GFP/mCherry-Tubulin interphase cells. pMT-CENP-A-GFP expression was induced for 2 hours with 10 μM CuSO_4_, washed and incubated for 22 h in conditional medium without CuSO_4_ before immunostaining with anti-CAL1 antibody (red). DNA (DAPI) is shown in grey. Scale bar: 2 μm. The graph shows the total CAL1 centromeric intensity per nucleus as % of control. Mean +/- SEM of 3 experiments (n>300 cells), Student’s t-test (***: p<0.001). **B**. Mitotic phenotypes of pMT-CENP-A-GFP/mCherry-Tubulin expressing cells. After 2 h pMT-CENP-A-GFP induction cells were imaged for 16 h and scored for their accuracy of mitosis: lagging (presence of lagging chromosomes during anaphase that will resolve before cytokinesis) or defective (formation of tripolar spindles, multinucleated cells). Mean +/- SEM n > 200 cells. Student’s t-test (*n*.*s*.: non-significant). **C**. Ndc80 kinetochore levels in pMT-CENP-A-GFP/mCherry-Tubulin mitotic cells. After 2 h pMT-CENP-A-GFP induction, fixed cells were stained with anti-Ndc80 antibody (red). DNA (DAPI) is shown in blue. Scale bar: 2 μm. The graph shows the total Ndc80 kinetochore intensity per cell as % of control. Only prometaphase cells were analyzed. Mean +/- SEM of 3 experiments (n>90 cells), Student’s t-test (***: p<0.001). **D**. Kinetochore-microtubule attachment assay. After 2 h pMT-CENP-A-GFP induction, cells were incubated with 20 μM MG132 for 1 h, and with 100 nM Taxol for 3 h. Cells were then fixed and the DNA counterstained with DAPI. Cells were scored into 2 categories: either “attached” when each kinetochore was attached to microtubules, or “> 1 unattached” when at least 1 kinetochore was not stably connected to microtubules. The graph shows the percentage of cells in each category as Mean +/- SEM, N = 2.
(TIF)

S5 Fig Related to Figs 4 and 5. **A**. Immunoblot showing CENP-A and Mad2 double knockdown efficiency in GFP-CENP-A/mCherry-Tubulin expressing cells using anti-CENP-A, anti-Mad2, and tubulin antibodies. **B**. Immunoblot showing BubR1 (top blot) or Mad2 (bottom blot) knockdown efficiency in SNAP-CENP-A expressing cells using anti-BubR1, anti-Mad2, and tubulin or H3 antibodies as loading controls. **C**. qPCR results showing mRNA levels after indicated knockdown in SNAP-CENP-A cells as percent of control. **D**. Immunofluorescence of SNAP-CENP-A expressing cells after MG132 or Nocodazole treatment. A Quench-Chase-Pulse experiment was performed to stain newly synthesized SNAP-CENP-A molecules (red). The 24 h chase was performed in the presence or absence of the indicated drug. Cells were stained with an anti-S10-phospho-H3 antibody (green) to identify mitotic cells. DNA (DAPI) is shown in grey. Scale bar: 2 μm. **E**. Quantification of D showing the SNAP-CENP-A centromeric intensity as % of control. Only phospho-H3 positive cells were analyzed. Mean +/- SEM of 3 experiments (n>90 cells), Student’s t-test (n.s = non-significant). **F**. Quantification showing the total SNAP-CAL1 centromeric intensity per nucleus as % of control after knockdown of the indicated proteins. Mean +/- SEM of 3 experiments (n>300 cells), Student’s t-test (**: p<0.01; n.s.: non-significant).
(TIF)

S6 Fig Related to Figs 6 and 7. **A**. Yeast two-hybrid interaction assay. Blue color reflects the interaction between the 2 proteins tested. **B**. Scheme showing CAL1 functional domains and its binding to its known partners. BD: binding domain. **C. *Left panel***. Coomassie showing purified His-CENP-A. ***Right panel***. Pulldown assay of GST or GST-Zw10 with His-CENP-A. **D-F**. Localization of GFP-Zw10 (**D**), GFP-Zwilch (**E**), GFP-ROD (**F**) during the cell cycle. Cells expressing each GFP-RZZ component concomitant with mCherry-Tubulin were fixed and stained with anti-GFP (green) and anti-CENP-A (red) antibodies. DNA (DAPI) is shown in blue. Scale bar: 2 μm.
(TIF)

S7 Fig Related to Fig 6. **A**. Immunofluorescence of SNAP-CAL1 expressing cells after Zw10 depletion. After 72 h dsRNA treatment, a Quench-Chase-Pulse experiment (scheme in Fig 1H) was performed to stain newly synthesized SNAP-CAL1 molecules (red). DNA (DAPI) is shown in blue. Scale bar: 2 μm. **B**. Quantification of A showing the total SNAP-CAL1 centromeric intensity per nucleus as % of control. Mean +/- SEM of 3 experiments (n>300 cells), Student’s t-test (**: p<0.01). **C**. SNAP Pulse-Chase experiment of SNAP-CENP-A expressing cells after Zw10 depletion. At day 3 of RNAi, cells were incubated with TMR-Star (P) to stain existing SNAP-CENP-A molecules (red), washed, and put back in culture (t0). After a 24 h chase, cells were fixed (t24). Note that no SNAP-Block was performed for this experiment. DNA (DAPI) is shown in grey. Scale bar: 2 μm. **D**. Quantification of C showing the total SNAP-CENP-A centromeric intensity per nucleus as % of t0. Mean +/- SEM of 3 experiments (n>300 cells), Student’s t-test (n.s = non-significant). **E**. FRAP of GFP-CENP-A in mitosis after 72 h of Zw10 depletion. GFP-CENP-A signal was partially (about 50–60%) bleached in prophase and cells were imaged until telophase. Time-lapse: 90 s. Scale bar: 2 μm. **F**. Quantification of E. The total GFP-CENP-A centromeric signal of at least 8 cells is displayed as Mean +/- SEM.
(TIF)

S8 Fig Related to Figs 7 and 8. **A**. qPCR results showing mRNA levels after indicated knockdowns in GFP-Zw10 cells as percent of control. **B-D**. Immunofluorescence with anti-GFP (green) and anti-CENP-A (B, red) or Scp105R (C-D, red) antibodies of GFP-Zw10/mCherry-Tubulin expressing cells after 96 h depletion of CAL1 (**B**), Bub1 (**C**) or Mis12 (**D**). DNA (DAPI) is shown in blue. Scale bar: 2 μm. **E**. Immunofluorescence with anti-Mis12 (green), anti-Spc105R (red) and anti-tubulin (grey) antibodies of S2 cells after 96 h of Zw10 depletion. DNA (DAPI) is shown in blue. Scale bar: 2 μm. **F**. Quantification of E showing the total Mis12 kinetochore intensity per mitotic cell. Mis12 fluorescence intensity at kinetochores was measured for each cell and normalized within one experiment before pooling measurements from at least 3 experiments per condition. Mean +/- SEM (n>90 cells), Student’s t-test (n.s.: non-significant). **G**. Immunofluorescence with anti-BubR1 (green) and anti-tubulin (grey) antibodies of S2 cells after 96 h Zw10 depletion. DNA (DAPI) is shown in blue. Scale bar = 2 μm. **H**. Quantification of G showing the total BubR1 kinetochore intensity per mitotic cell. BubR1 fluorescence intensity at kinetochores was measured for each cell and normalized within one experiment before pooling measurements from at least 3 experiments per condition. Mean +/- SEM (n>90 cells), Student’s t-test (n.s.: non-significant). **I**. Model of the potential association of SAC proteins with the Drosophila kinetochore. We propose two branches of recruitment of SAC proteins in Drosophila cells: the described dependence of the Bub proteins on Spc105R and CENP-C on one hand and the newly suggested association of the RZZ (and therefore the Mad proteins) through direct interaction of Zw10 with CAL1.
(TIF)

S1 Video (Related to Fig 2C). SNAP-CENP-A/mCherry-tubulin expressing cells containing inducible pMT-CAL1-V5. No pMT-CAL1-V5 induction. SNAP-CENP-A molecules were quenched before the addition of 0.5 μM SNAP-640 dye. Imaging was performed for 16 h on a Deltavision microscope (60X, Bin 2), at 25°C. Time-lapse 15 min. Scale bar: 5 μm.
(AVI)

S2 Video (Related to Fig 2C). SNAP-CENP-A/mCherry-tubulin expressing cells containing inducible pMT-CAL1-V5. pMT-CAL1-V5 expression was induced for 24 h with 100 μM CuSO_4_. SNAP-CENP-A molecules were quenched before the addition of 0.5 μM SNAP-640 dye. Imaging was performed for 16 h on a Deltavision microscope (60X, Bin 2), at 25°C. Time-lapse 15 min. Scale bar: 5 μm.
(AVI)

S3 Video (Related to Fig 2F). H2B-GFP/mCherry-tubulin expressing cells containing inducible pMT-CAL1-V5. No pMT-CAL1-V5 induction. Imaging was performed for 16 h on a Deltavision microscope (60X, Bin 2), at 25°C. Time-lapse 3 min. Scale bar: 5 μm.
(AVI)

S4 Video (Related to Fig 2F). H2B-GFP/mCherry-tubulin expressing cells containing inducible pMT-CAL1-V5. pMT-CAL1-V5 expression was induced with 100 μM CuSO_4_ for 24 h prior to imaging. Imaging was performed for 16 h on a Deltavision microscope (60X, Bin 2), at 25°C. Time-lapse 3 min. Scale bar: 5 μm.
(AVI)

S5 Video (Related to Fig 3E). pMT-CENP-A-GFP/mCherry-tubulin expressing cells. Visible CENP-A-GFP results from leaky metallothionein promoter. Imaging was performed for 16 h on a Deltavision microscope (40X, Bin 2), at 25 °C. Time-lapse 3 min. Scale bar: 5 μm.
(AVI)

S6 Video (Related to Fig 3E). pMT-CENP-A-GFP/mCherry-tubulin expressing cells. pMT-CENP-A-GFP expression was induced for 2 h with 10 μM CuSO_4_ then washed 3 times before being placed in Ibidi chamber. Imaging was performed for 16 h on a Deltavision microscope (40X, Bin 2), at 25°C. Time-lapse 3 min. Scale bar: 5 μm.
(AVI)

S7 Video (Related to Fig 4C). GFP-CENP-A/mCherry-Tubulin expressing cells after Ctrl dsRNA treatment (72 h). Imaging was performed for 16 h on a Deltavision microscope (40X, Bin 2), at 25°C. Time-lapse 3 min. Scale bar: 5 μm.
(AVI)

S8 Video 6 (Related to Fig 4C). GFP-CENP-A/mCherry-Tubulin expressing cells after CENP-A dsRNA treatment (72 h). Imaging was performed for 16 h on a Deltavision microscope (40X, Bin 2), at 25°C. Time-lapse 3 min. Scale bar: 5 μm.
(AVI)

S9 Video (Related to Fig 6D). H2B-GFP/mCherry-tubulin expressing cells after Ctrl dsRNA treatment (72 h). Imaging was performed for 16 h on a Deltavision microscope (60X, Bin 2), at 25°C. Time-lapse 3 min. Scale bar: 5 μm.
(AVI)

S10 Video (Related Fig 6D). H2B-GFP/mCherry-tubulin expressing cells after Zw10 dsRNA treatment (72 h). Imaging was performed for 16 h on a Deltavision microscope (60X, Bin 2), at 25°C. Time-lapse 3 min. Scale bar: 5 μm.
(AVI)
