# Supplementary figures and images for: The checkpoint protein Zw10 connects CAL1-dependent CENP-A centromeric loading and mitosis duration in Drosophila cells
Source: PLoS Genet. 2019 Sep 25;15(9):e1008380. doi: 10.1371/journal.pgen.1008380 (PMC6779278; doi:10.1371/journal.pgen.1008380)

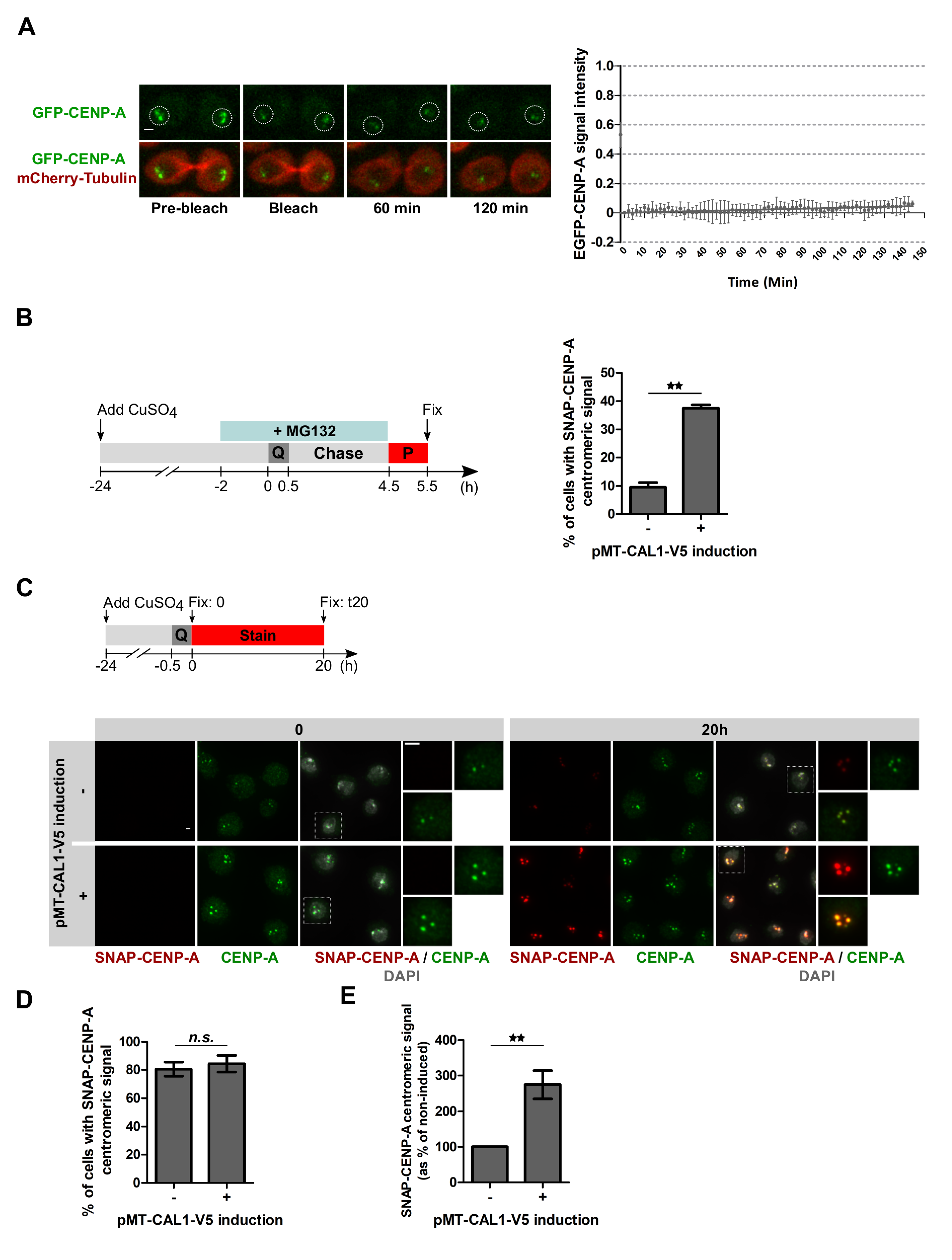

Supplement: S1 Fig — A. FRAP of GFP-CENP-A in G1 phase. Cells expressing GFP-CENP-A and mCherry-Tubulin were followed through mitosis, GFP-CENP-A signal was partially bleached in early G1 and cells were further imaged for > 2 h. Time-lapse: 6 min. Scale bar: 2 μm. The total GFP-CENP-A centromeric signal of 6 cells is shown as mean +/- SEM. B. SNAP Quench-Chase-Pulse experiment in MG132-treated cells. After 24 h induction of pMT-CAL1-V5, cells were incubated with MG132 for 2 h to arrest cells in mitosis prior to the SNAP-block. A 4-h chase was performed in presence of MG132 to allow synthesis and incorporation of new SNAP-CENP-A into centromeres before staining with SNAP-Si647 and fixation. The graph shows the percentage of cells with SNAP-CENP-A at centromeres. C. Immunofluorescence of SNAP-CENP-A in control (non-induced) or induced (24 h) pMT-CAL1-V5 cells. The cells were incubated with SNAP-Block, washed, incubated with SNAP-640 dye for 20 h before immunostaining with an anti-CENP-A antibody (green), or taken directly after block (0 h), and stained with SNAP-640 dye for 15 min to check the efficiency of the block. DNA (DAPI) is shown in grey. Scale bar: 2 μm. D. Quantification of C showing the percentage of cells positive for centromeric SNAP-CENP-A staining. E. Quantification of C showing the total SNAP-CENP-A centromeric intensity per nucleus as % of control. All graphs show Mean +/- SEM of 3 experiments (n>300 cells), Student’s t-test (n.s.: non-significant; *: p<0.05; **: p<0.01). (TIF) [file pgen.1008380.s001.tif]

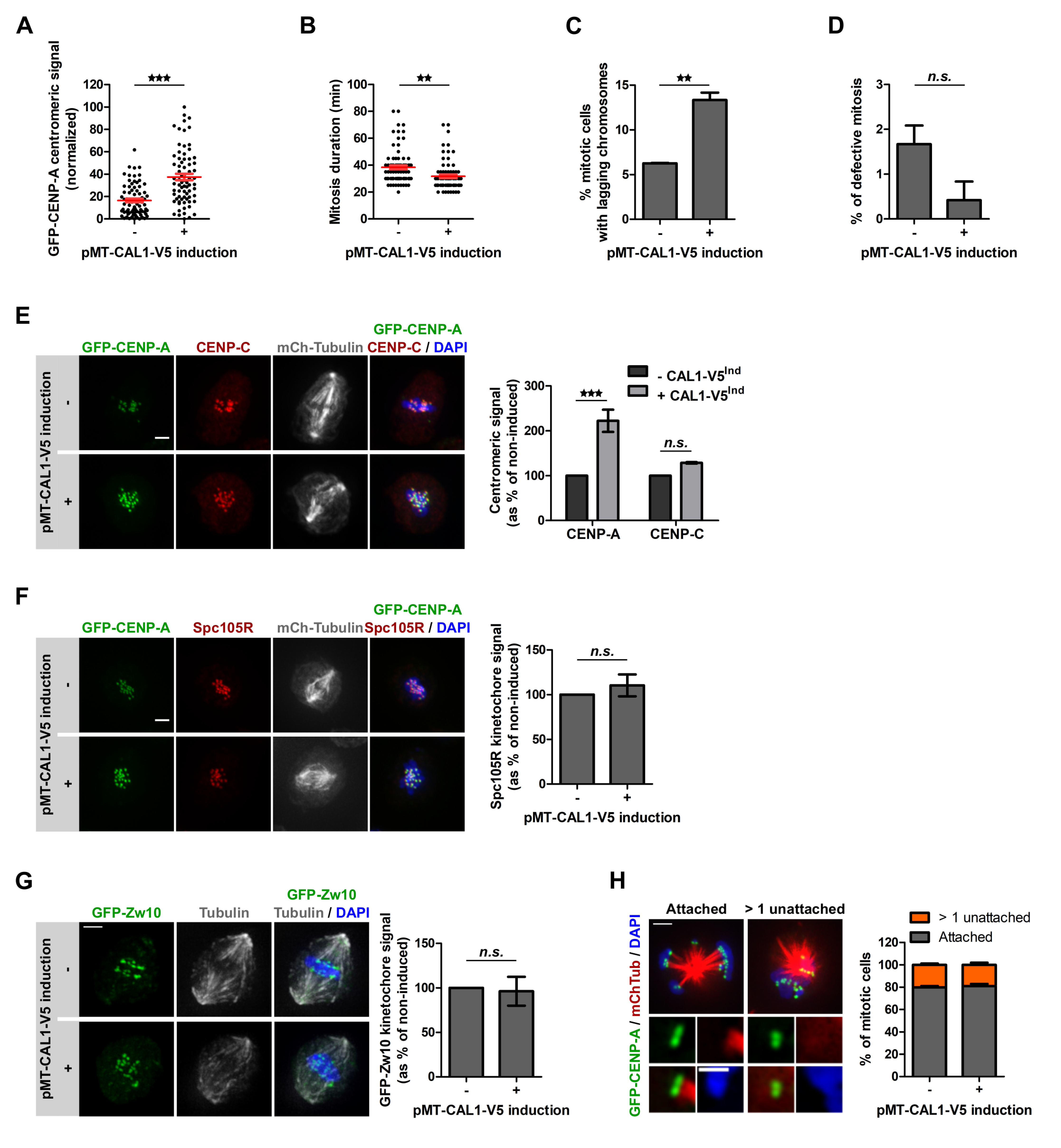

Supplement: S2 Fig — A. Quantification showing GFP-CENP-A centromeric signal intensity per nucleus at t0 of time-lapse imaging with or without pMT-CAL1-V5 induction. Mean +/- SEM, n>80 cells. Student’s t-test (***: p<0.001). Data from 2 experiments were normalized and combined. B. Time-lapse imaging of GFP-CENP-A/mCherry-tubulin expressing cells with or without prior pMT-CAL1-V5 induction (100 μM CuSO4, 24 h). Imaging: 16 h. Time-lapse: 3 min. Scale bar: 2 μm. C-D Mitotic phenotypes of CAL1 overexpression. pMT-CAL1-V5 expression was induced for 24 h in H2B-GFP/mCherry-Tubulin cells. Cells were imaged for 16 h and scored for the accuracy of mitosis: lagging (presence of lagging chromosomes during anaphase that will resolve before cytokinesis)(C) or defective (formation of tripolar spindles, multinucleated cells)(D). Mean +/- SEM n > 200 cells. Student’s t-test (n.s.: non-significant). E-G. Amount of kinetochore proteins recruited during mitosis in the presence or absence of CAL1 overexpression. pMT-CAL1-V5 expression was induced for 24 h in GFP-CENP-A/mCherry-Tubulin cells (E-F) or GFP-Zw10 expressing cells (G). Fixed cells were stained with anti-CENP-C (E, red), anti-Spc105R (F, red) or anti-tubulin (G, grey). DNA (DAPI) is shown in blue. Scale bar: 2 μm. Kinetochore signal intensity of the indicated proteins is shown as % of control. Only prometaphase cells were analyzed. Mean +/- SEM of 3 experiments (n > 90 cells). Student’s t-test (n.s.: non-significant). H. Kinetochore-microtubule attachment assay. After 24 h pMT-CAL1-V5 induction, GFP-CENP-A/mCherry-Tubulin cells were incubated with 20 μM MG132 for 1 h, then with 100 nM Taxol for 3 h. The fixed cells were scored into 2 categories: either “attached” when each kinetochore was attached to microtubules, or “> 1 unattached” when at least 1 kinetochore was not stably connected to microtubules. The graph shows the percentage of cells in each category as Mean +/- SEM, N = 2. (TIF) [file pgen.1008380.s002.tif]

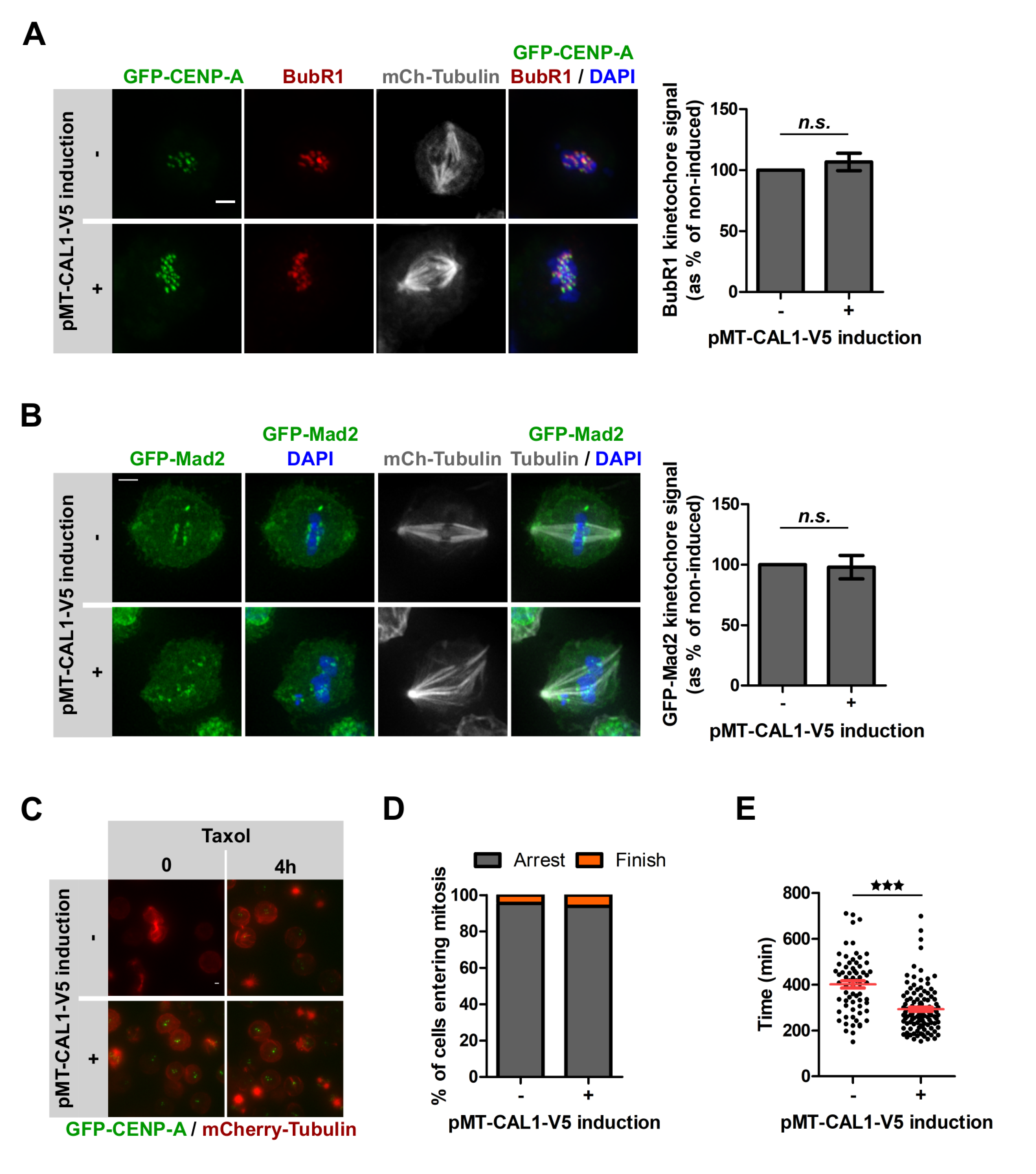

Supplement: S3 Fig — A-B. Amount of SAC proteins recruited to the kinetochore during mitosis in the presence or absence of CAL1 overexpression. pMT-CAL1-V5 expression was induced for 24 h in GFP-CENP-A/mCherry-Tubulin cells (A) or GFP-Mad2/mCherry-Tubulin (B) expressing cells. Fixed cells were stained with anti-BubR1 (A, red). DNA (DAPI) is shown in blue. Scale bar: 2 μm. Kinetochore signal intensity of the indicated proteins is shown as % of control. Only prometaphase cells were analyzed. Mean +/- SEM of 3 experiments (n > 90 cells). Student’s t-test (n.s.: non-significant). C. Stills from time-lapse imaging experiments showing GFP-CENP-A/mCherry-Tubulin cells with or without prior pMT-CAL1-V5 induction (24 h) imaged immediately after the addition of 1 μM Taxol and after 4 h. Imaging: 16 h. Scale bar: 2 μm. D. Quantification of C showing the percentage of cells that arrest (‘arrest’) in response to Taxol treatment or arrest and restart to finish mitosis (‘finish’). E. Quantification of time-lapse imaging showing the time taxol-treated pMT-CAL1-V5-overexpressing cells spend in an arrested state before either reverting to G2-like state or proceeding through mitosis. Mean +/- SEM, n>100 cells. Student’s t-test (***: p<0.001). (TIF) [file pgen.1008380.s003.tif]

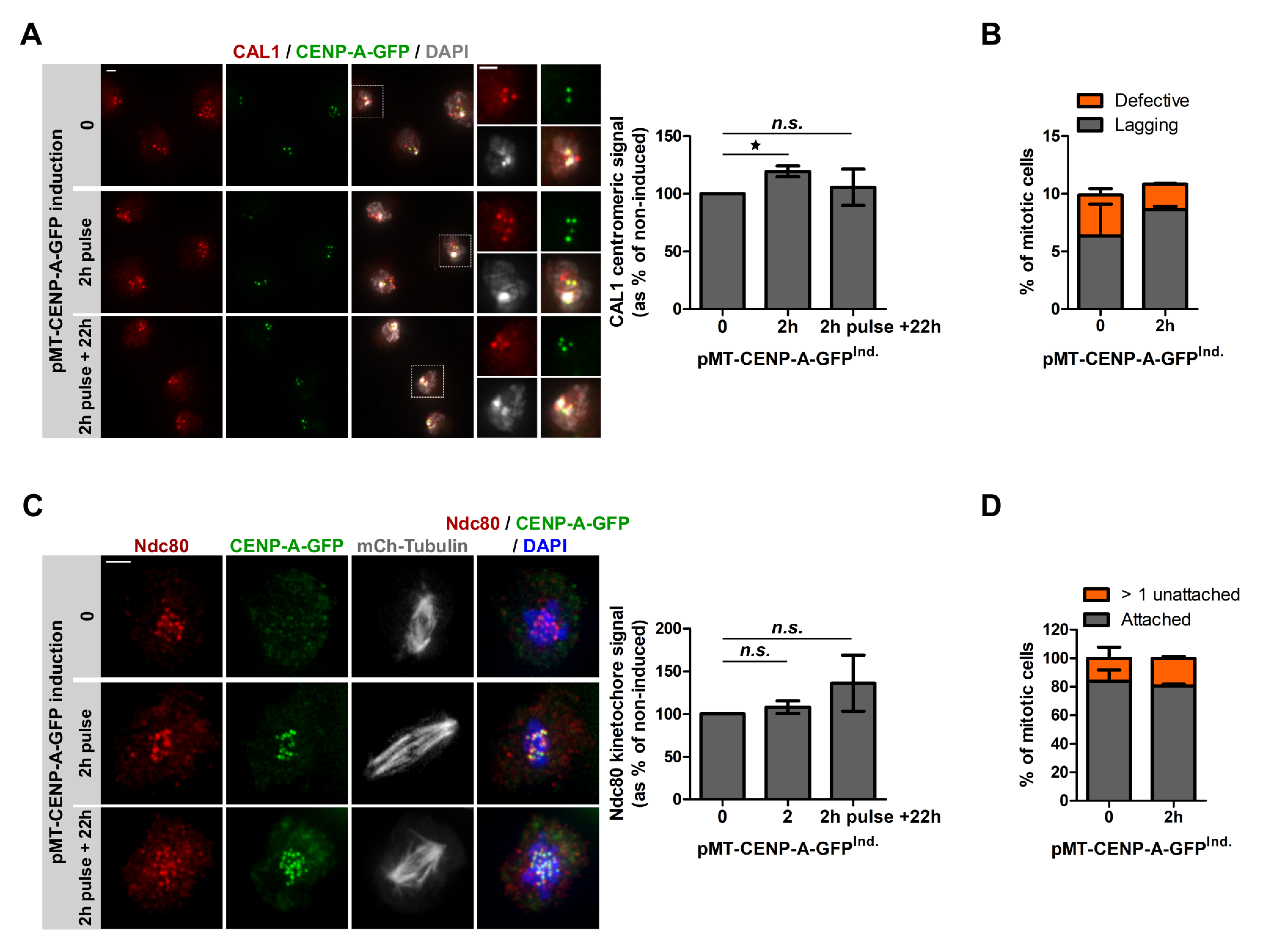

Supplement: S4 Fig — A. CAL1 centromeric levels in pMT-CENP-A-GFP/mCherry-Tubulin interphase cells. pMT-CENP-A-GFP expression was induced for 2 hours with 10 μM CuSO4, washed and incubated for 22 h in conditional medium without CuSO4 before immunostaining with anti-CAL1 antibody (red). DNA (DAPI) is shown in grey. Scale bar: 2 μm. The graph shows the total CAL1 centromeric intensity per nucleus as % of control. Mean +/- SEM of 3 experiments (n>300 cells), Student’s t-test (***: p<0.001). B. Mitotic phenotypes of pMT-CENP-A-GFP/mCherry-Tubulin expressing cells. After 2 h pMT-CENP-A-GFP induction cells were imaged for 16 h and scored for their accuracy of mitosis: lagging (presence of lagging chromosomes during anaphase that will resolve before cytokinesis) or defective (formation of tripolar spindles, multinucleated cells). Mean +/- SEM n > 200 cells. Student’s t-test (n.s.: non-significant). C. Ndc80 kinetochore levels in pMT-CENP-A-GFP/mCherry-Tubulin mitotic cells. After 2 h pMT-CENP-A-GFP induction, fixed cells were stained with anti-Ndc80 antibody (red). DNA (DAPI) is shown in blue. Scale bar: 2 μm. The graph shows the total Ndc80 kinetochore intensity per cell as % of control. Only prometaphase cells were analyzed. Mean +/- SEM of 3 experiments (n>90 cells), Student’s t-test (***: p<0.001). D. Kinetochore-microtubule attachment assay. After 2 h pMT-CENP-A-GFP induction, cells were incubated with 20 μM MG132 for 1 h, and with 100 nM Taxol for 3 h. Cells were then fixed and the DNA counterstained with DAPI. Cells were scored into 2 categories: either “attached” when each kinetochore was attached to microtubules, or “> 1 unattached” when at least 1 kinetochore was not stably connected to microtubules. The graph shows the percentage of cells in each category as Mean +/- SEM, N = 2. (TIF) [file pgen.1008380.s004.tif]

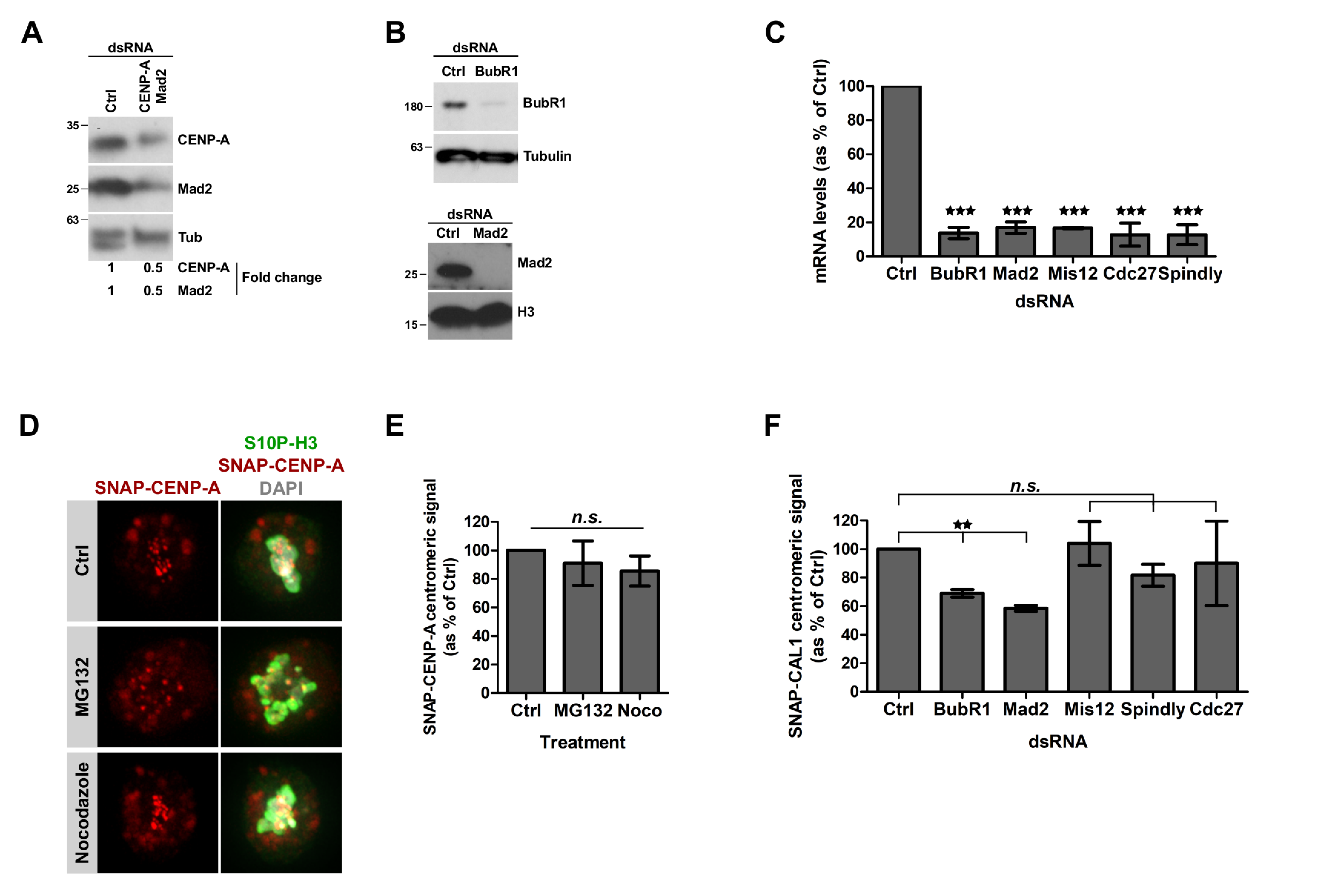

Supplement: S5 Fig — A. Immunoblot showing CENP-A and Mad2 double knockdown efficiency in GFP-CENP-A/mCherry-Tubulin expressing cells using anti-CENP-A, anti-Mad2, and tubulin antibodies. B. Immunoblot showing BubR1 (top blot) or Mad2 (bottom blot) knockdown efficiency in SNAP-CENP-A expressing cells using anti-BubR1, anti-Mad2, and tubulin or H3 antibodies as loading controls. C. qPCR results showing mRNA levels after indicated knockdown in SNAP-CENP-A cells as percent of control. D. Immunofluorescence of SNAP-CENP-A expressing cells after MG132 or Nocodazole treatment. A Quench-Chase-Pulse experiment was performed to stain newly synthesized SNAP-CENP-A molecules (red). The 24 h chase was performed in the presence or absence of the indicated drug. Cells were stained with an anti-S10-phospho-H3 antibody (green) to identify mitotic cells. DNA (DAPI) is shown in grey. Scale bar: 2 μm. E. Quantification of D showing the SNAP-CENP-A centromeric intensity as % of control. Only phospho-H3 positive cells were analyzed. Mean +/- SEM of 3 experiments (n>90 cells), Student’s t-test (n.s = non-significant). F. Quantification showing the total SNAP-CAL1 centromeric intensity per nucleus as % of control after knockdown of the indicated proteins. Mean +/- SEM of 3 experiments (n>300 cells), Student’s t-test (**: p<0.01; n.s.: non-significant). (TIF) [file pgen.1008380.s005.tif]

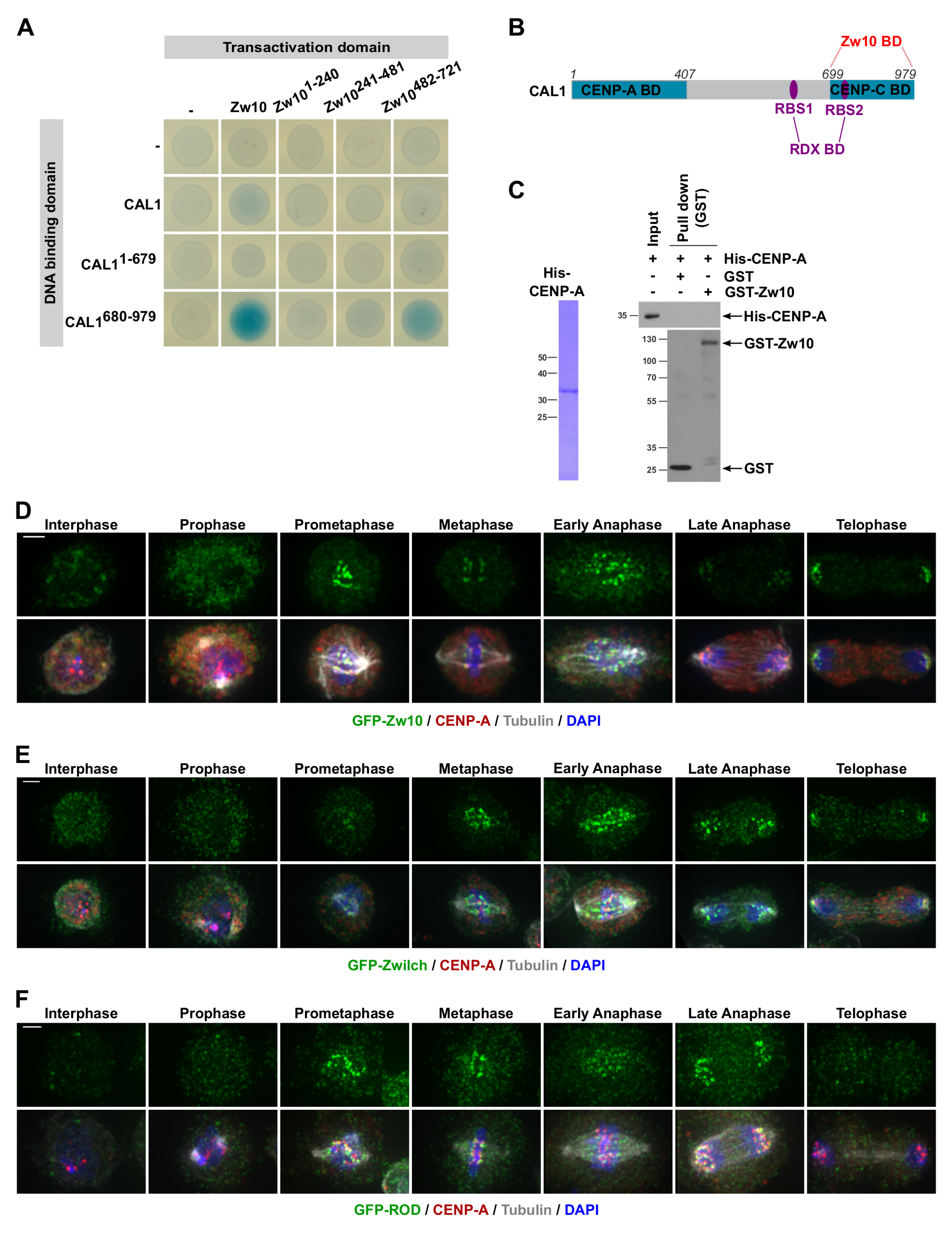

Supplement: S6 Fig — A. Yeast two-hybrid interaction assay. Blue color reflects the interaction between the 2 proteins tested. B. Scheme showing CAL1 functional domains and its binding to its known partners. BD: binding domain. C. Left panel. Coomassie showing purified His-CENP-A. Right panel. Pulldown assay of GST or GST-Zw10 with His-CENP-A. D-F. Localization of GFP-Zw10 (D), GFP-Zwilch (E), GFP-ROD (F) during the cell cycle. Cells expressing each GFP-RZZ component concomitant with mCherry-Tubulin were fixed and stained with anti-GFP (green) and anti-CENP-A (red) antibodies. DNA (DAPI) is shown in blue. Scale bar: 2 μm. (TIF) [file pgen.1008380.s006.tif]

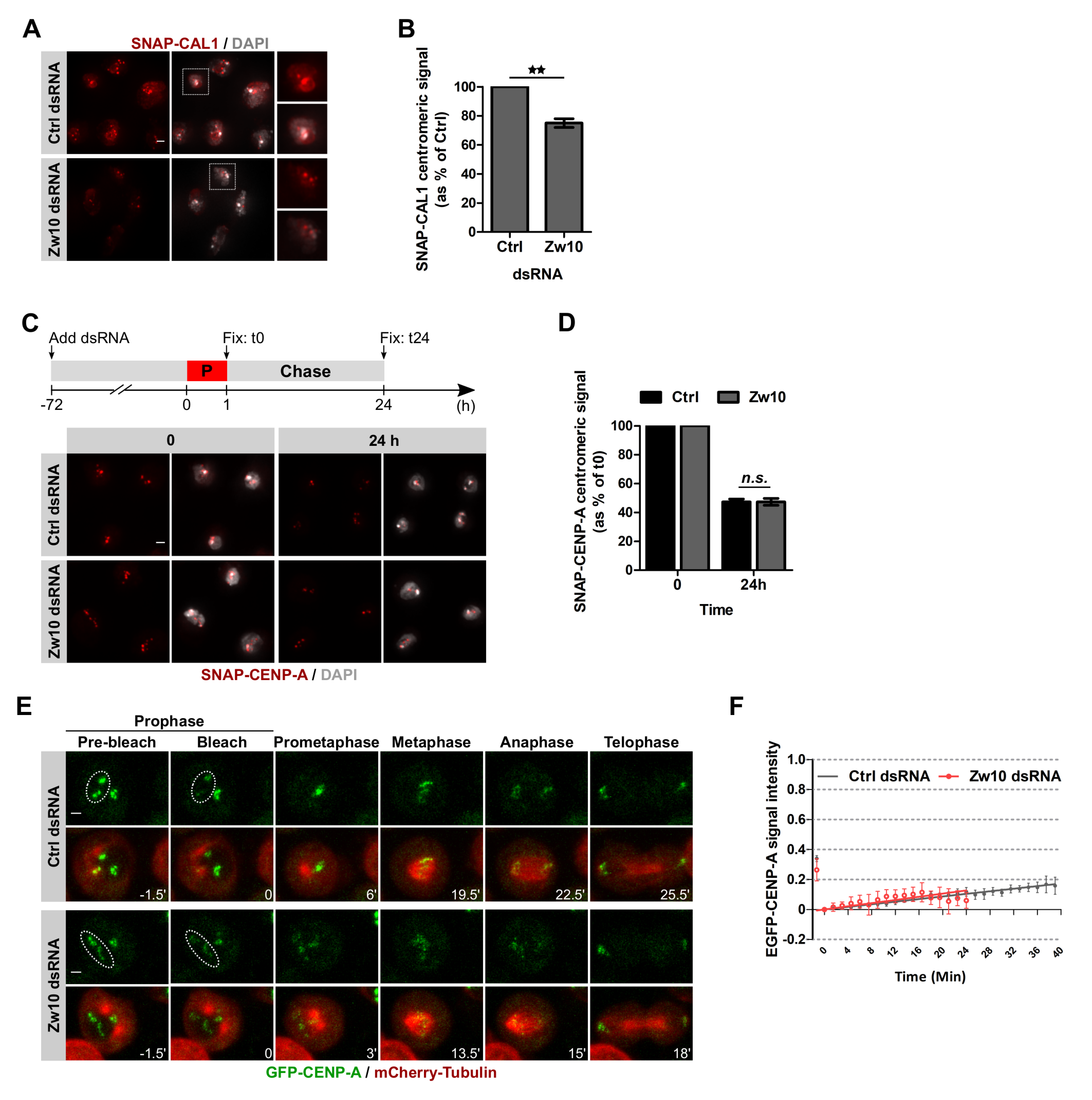

Supplement: S7 Fig — A. Immunofluorescence of SNAP-CAL1 expressing cells after Zw10 depletion. After 72 h dsRNA treatment, a Quench-Chase-Pulse experiment (scheme in Fig 1H) was performed to stain newly synthesized SNAP-CAL1 molecules (red). DNA (DAPI) is shown in blue. Scale bar: 2 μm. B. Quantification of A showing the total SNAP-CAL1 centromeric intensity per nucleus as % of control. Mean +/- SEM of 3 experiments (n>300 cells), Student’s t-test (**: p<0.01). C. SNAP Pulse-Chase experiment of SNAP-CENP-A expressing cells after Zw10 depletion. At day 3 of RNAi, cells were incubated with TMR-Star (P) to stain existing SNAP-CENP-A molecules (red), washed, and put back in culture (t0). After a 24 h chase, cells were fixed (t24). Note that no SNAP-Block was performed for this experiment. DNA (DAPI) is shown in grey. Scale bar: 2 μm. D. Quantification of C showing the total SNAP-CENP-A centromeric intensity per nucleus as % of t0. Mean +/- SEM of 3 experiments (n>300 cells), Student’s t-test (n.s = non-significant). E. FRAP of GFP-CENP-A in mitosis after 72 h of Zw10 depletion. GFP-CENP-A signal was partially (about 50–60%) bleached in prophase and cells were imaged until telophase. Time-lapse: 90 s. Scale bar: 2 μm. F. Quantification of E. The total GFP-CENP-A centromeric signal of at least 8 cells is displayed as Mean +/- SEM. (TIF) [file pgen.1008380.s007.tif]

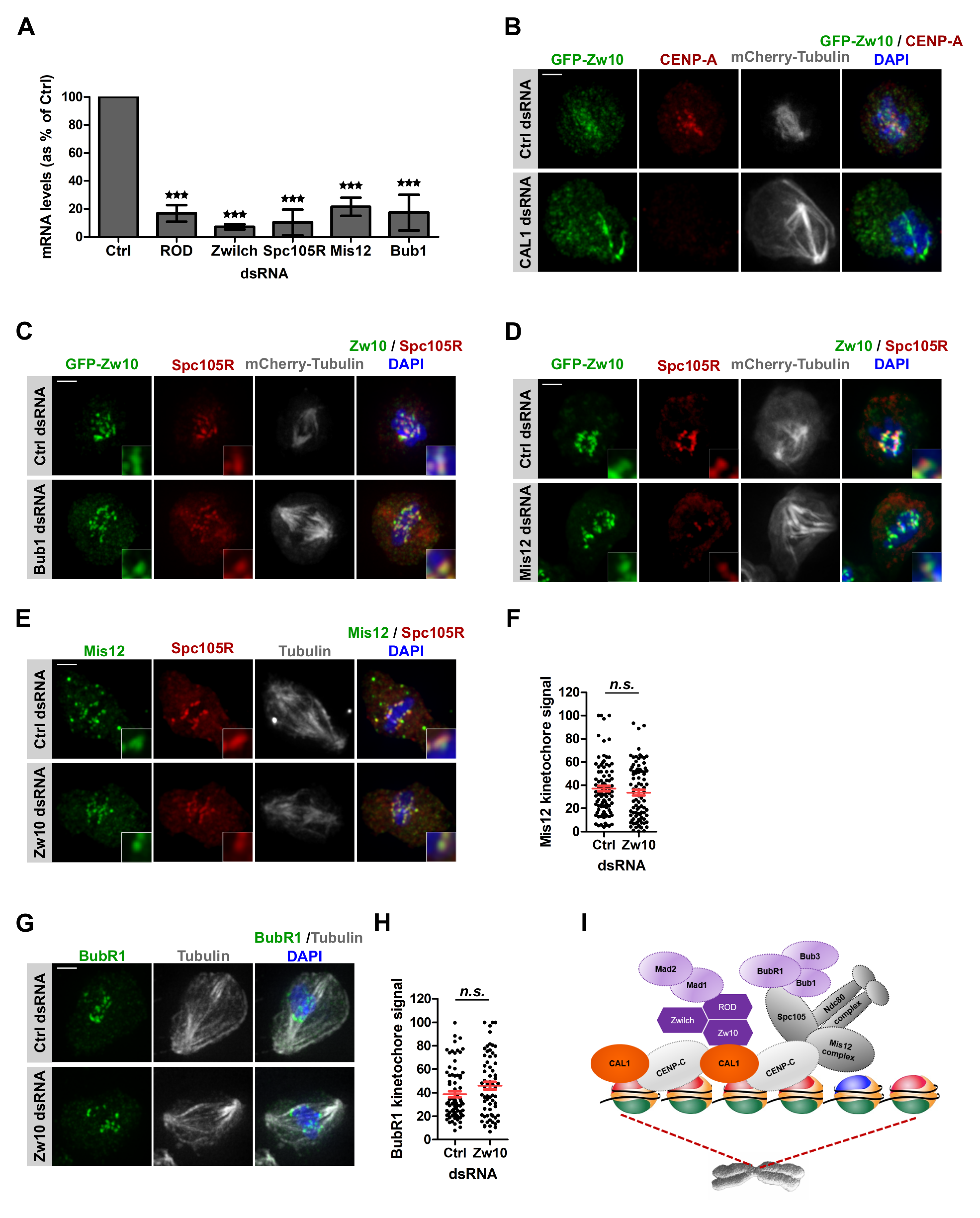

Supplement: S8 Fig — A. qPCR results showing mRNA levels after indicated knockdowns in GFP-Zw10 cells as percent of control. B-D. Immunofluorescence with anti-GFP (green) and anti-CENP-A (B, red) or Scp105R (C-D, red) antibodies of GFP-Zw10/mCherry-Tubulin expressing cells after 96 h depletion of CAL1 (B), Bub1 (C) or Mis12 (D). DNA (DAPI) is shown in blue. Scale bar: 2 μm. E. Immunofluorescence with anti-Mis12 (green), anti-Spc105R (red) and anti-tubulin (grey) antibodies of S2 cells after 96 h of Zw10 depletion. DNA (DAPI) is shown in blue. Scale bar: 2 μm. F. Quantification of E showing the total Mis12 kinetochore intensity per mitotic cell. Mis12 fluorescence intensity at kinetochores was measured for each cell and normalized within one experiment before pooling measurements from at least 3 experiments per condition. Mean +/- SEM (n>90 cells), Student’s t-test (n.s.: non-significant). G. Immunofluorescence with anti-BubR1 (green) and anti-tubulin (grey) antibodies of S2 cells after 96 h Zw10 depletion. DNA (DAPI) is shown in blue. Scale bar = 2 μm. H. Quantification of G showing the total BubR1 kinetochore intensity per mitotic cell. BubR1 fluorescence intensity at kinetochores was measured for each cell and normalized within one experiment before pooling measurements from at least 3 experiments per condition. Mean +/- SEM (n>90 cells), Student’s t-test (n.s.: non-significant). I. Model of the potential association of SAC proteins with the Drosophila kinetochore. We propose two branches of recruitment of SAC proteins in Drosophila cells: the described dependence of the Bub proteins on Spc105R and CENP-C on one hand and the newly suggested association of the RZZ (and therefore the Mad proteins) through direct interaction of Zw10 with CAL1. (TIF) [file pgen.1008380.s008.tif]
